# ALK4/5-dependent TGF-β signaling contributes to the crosstalk between neurons and microglia following axonal lesion

**DOI:** 10.1038/s41598-019-43328-x

**Published:** 2019-05-03

**Authors:** Antonella Raffo-Romero, Tanina Arab, Christelle Van Camp, Quentin Lemaire, Maxence Wisztorski, Julien Franck, Soulaimane Aboulouard, Francoise Le Marrec-Croq, Pierre-Eric Sautiere, Jacopo Vizioli, Michel Salzet, Christophe Lefebvre

**Affiliations:** 1 University Lille, Inserm, U-1192 - Laboratoire Protéomique, Réponse Inflammatoire et Spectrométrie de Masse-PRISM, F-59000 Lille, France; 2 EURON – European Graduate School of Neuroscience, Maastricht, The Netherlands

**Keywords:** Proteomics, Cellular neuroscience, Microglia

## Abstract

Neuronal activity is closely influenced by glia, especially microglia which are the resident immune cells in the central nervous system (CNS). Microglia in medicinal leech are the only cells able to migrate to the injury site within the 24 hours post-lesion. The microglia-neuron interactions constitute an important mechanism as there is neither astrocyte nor oligodendrocyte in the leech CNS. Given that axonal sprouting is impaired when microglia recruitment is inhibited, the crosstalk between microglia and neurons plays a crucial role in neuroprotection. The present results show that neurons and microglia both use ALK4/5 (a type of TGF-β receptor) signaling in order to maintain mutual exchanges in an adult brain following an axonal injury. Indeed, a TGF-β family member (nGDF) is immediately released by injured axons contributing to the early recruitment of ALK4/5^+^ microglia to the lesion site. Surprisingly, within the following hours, nGDF from microglia activates ALK4/5^+^ neurons to maintain a later microglia accumulation in lesion. Taken together, the results demonstrate that ALK4/5 signaling is essential throughout the response to the lesion in the leech CNS and gives a new insight in the understanding of this pathway. This latter is an important signal contributing to a correct sequential mobilization over time of microglia recruitment leading to axon regeneration.

## Introduction

Many activities of the Central Nervous System (CNS) are correlated to the neuro-inflammatory state. It is a complex mechanism increasingly considered but still poorly understood because immune processes are diverse and dependent on the cell environment. Importantly, several recent studies demonstrate that microglia (the resident immune cells of the brain having a myeloid origin) can act both as a cause and a consequence in the regulation of inflammatory events associated with neurodegenerative diseases^1^. In physiological conditions, microglia cells have an immunosurveillance status to ensure CNS homeostasis. In trauma, they change their stellate-shaped morphology to adopt an amoeboid conformation^2^. These brain resident cells are really important sentinels constituting a first line of response to injury or inflammatory processes. They strongly interact with other brain cells regulating neural circuits and synaptic transmission^3,4^. Microglia and macrophages are myeloid cells respectively from primitive or definitive hematopoiesis^5^. *In vivo* discrimination of resident microglia and macrophages functions remains difficult in the neuro-inflammatory balance^6^. The present report tackles this issue by using a complementary model, where only resident microglia move towards the lesioned tissue.

**Figure 1:**
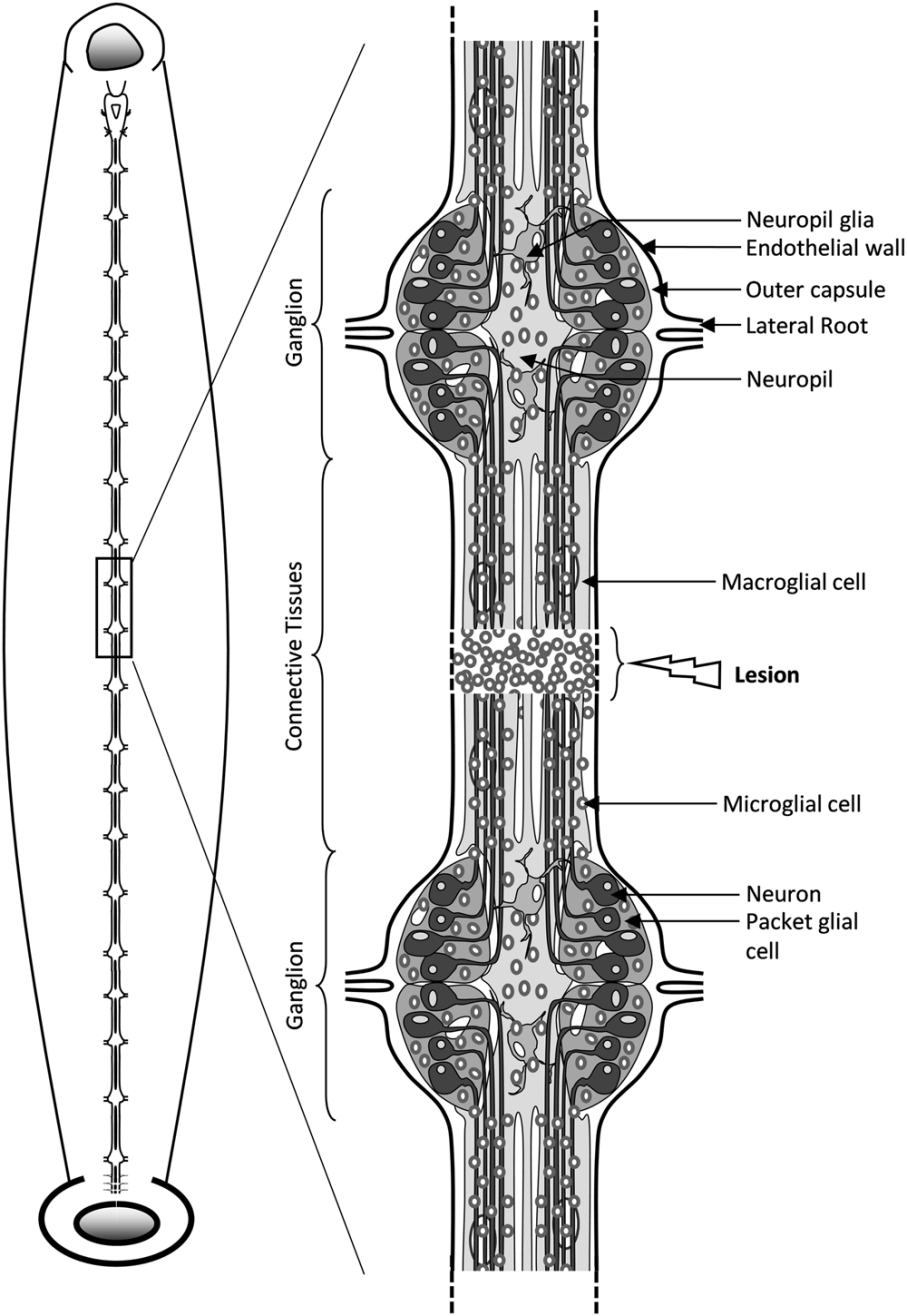
Diagram of Leech CNS. Left diagram shows the general structure of the nervous system in a dorsal view. Fused cerebral ganglia and fused caudal ganglia are above and below respectively. Right diagram shows a close-up of a fragment of 2 ganglia with connective fiber between them. The structure of a ganglion shows four of the six packet glial cells enveloping neuron cell bodies, surrounded by microglial cells. The projections of the neuronal axons pass through the neuropil and are prolonged into connectives. Microglial cells are distributed in ganglia and connectives fibers. The neuropil lies dorsomedially and contains two macroglial cells. Likewise, it is represented a lesion and a microglia accumulation following an experimental injury. The nervous system is enclosed in the outer capsule which is covered on the outside by a visceral layer of the endothelium (lining the ventral blood sinus).

The nerve cord from the medicinal leech (*Hirudo medicinalis*) is an excellent model in this regard. In the medicinal leech, microglial cells are also positive for iba1 marker as its mammalian counterparts. They progressively change their morphology from ramified to an amoeboid shape so that they move to the lesion without any blood cell infiltration^7,8^. Taking into account the lack of infiltration, blood cells could participate to the glial scar formation around endothelial cells and outside the CNS^9^ while microglia promote directly a regenerative process^10,11^. Microglia in leech are the only cells able to migrate to an injury site (Fig. 1) depending on chemotactic signals including ATP, C1q, EMAPII and Interleukin-16^12–17^. This recruitment massively occurs within the 24 hours post-lesion^18,19^. Interestingly, some authors have shown that when *in vivo* microglial accumulation is delayed by aberrant concentration of ATP, the axonal regrowth is consequently slowed down demonstrating that the recruitment of microglia is crucial to initiate the axonal sprouting^20^. The microglia-neuron interactions constitute an important mechanism as there is neither astrocyte nor oligodendrocyte in the leech CNS. The leech CNS contains microglia in ganglia as well as in their connective tissues (Fig. 1). All neuronal cell bodies are located in ganglia and most project their axons into the connective tissues^21^. Thus, ganglionic and connective microglia subpopulations can respectively interact with neuronal cell bodies or axons. Moreover, the tubular structure of the nerve chain makes possible to carry out a specific mechanical lesion in the middle of connectives to damage only the axons, keeping neuronal cell bodies intact. Because neuronal survival is not affected, it is possible in the leech CNS to study the mechanisms leading to the axonal sprouting and synaptogenesis. Consequently, elucidating the molecular processes involved in the crosstalk between neurons and microglia in leech can give a new insight in the refinement of a microenvironment-dependent response. On an evolutionary plan, the leech CNS gives the opportunity to understand the privileged interactions between neurons and microglia.

In this context, recent studies in murine microglia show that distinct tissue environments can induce high expression of *tgfbr1* and *tgfbr2* mRNAs, respectively coding for TGF-β type I (*alias* Activin receptor Like Kinase 5 or ALK5) and TGF-β type II receptors^22^. Importantly, TGF-β signaling is an essential driver of the development, maintenance and maturation of microglia populations^22–24^. In fact, the absence of TGF-β type II results in changes in microglial morphology and elevated expression of CD45. Both indicate a transformation to a blood macrophage profile^25^. Thus, the expression of TGF-β receptors is a specific signature of resident microglia not shared by blood macrophages. Given that TGF-β receptors regulate neuronal development^26,27^, we aim to better understand the roles played by TGF-β signaling in the crosstalk between microglia and neurons. In this report, we use an axonal lesion system in the connectives of the leech CNS. It involves the recruitment of microglia subpopulations over time. After the characterization of a TGF-β type I receptor in the leech microglia and neurons, its involvement in the time-course of microglia recruitment was evaluated following the axonal lesion.

## Results

### A TGF-β type I receptor is expressed in microglia cells and neurons

The analysis of leech-derived databases revealed the presence of a putative sequence coding for a *tgfbr1* orthologue. We characterized a TGF-β family type I receptor in the leech (Genbank Accession Number MH346327) after confirmation by RACE-PCR amplifications using specific primers. This protein presented a high amino acid sequence homology to the human TGF-β type I receptor (*alias* Activin receptor Like Kinase 5 or ALK5, UniProtKB P36897) and Activin type I receptor (*alias* ALK4, UniProtKB P36896) with respectively a 52% and 53% sequence identity (64% and 65% sequence similarity). The same level of sequence similarity was observed when we compared with the counterparts from other mammalian species.

**Figure 2:**
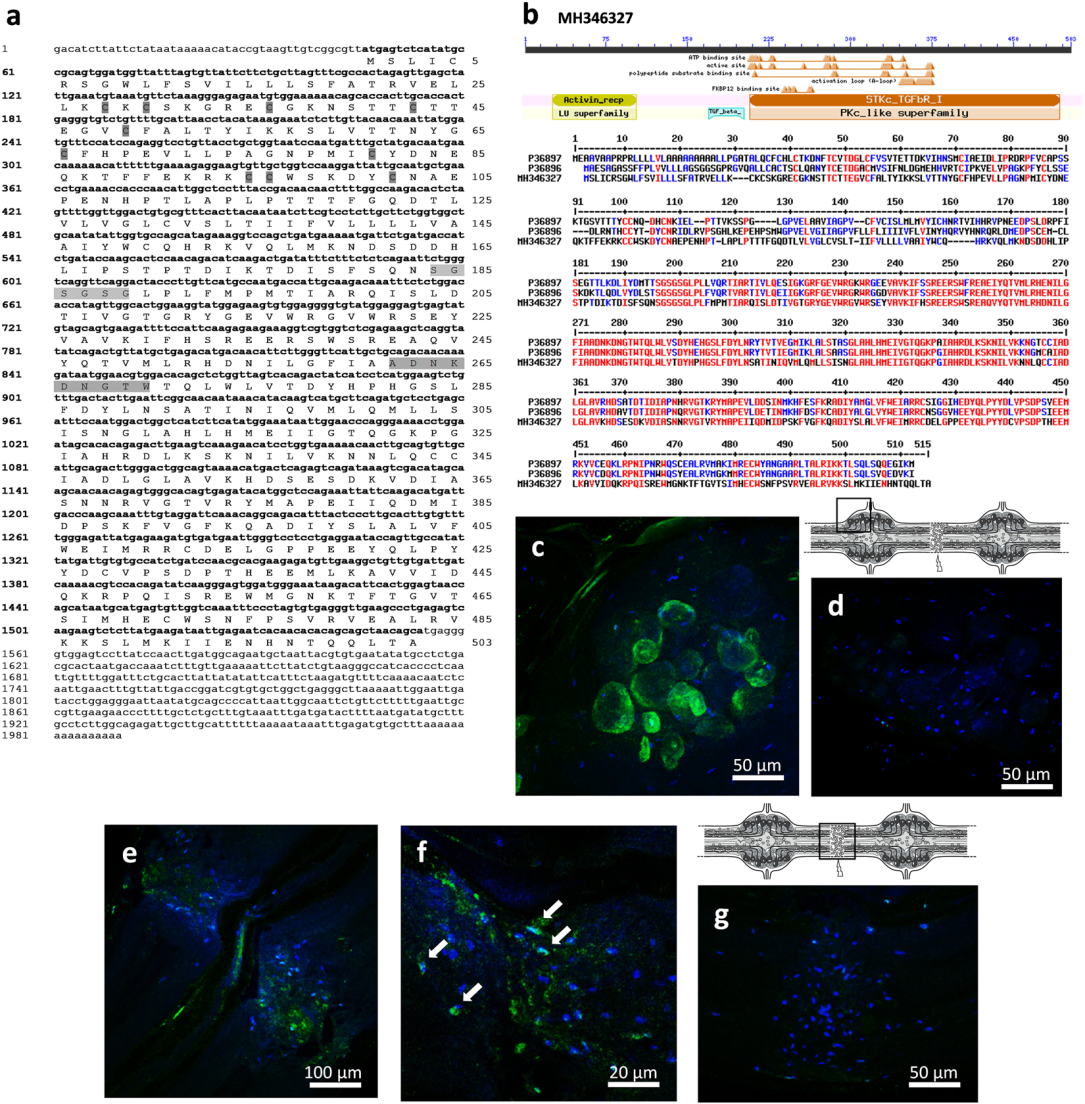
Molecular characterization of ALK4/5: a TGF-β family type I receptor (TGF-β R1). (**a**) Nucleotide and amino acid sequences of leech ALK4/5. The numbers of nucleotides are indicated in left and of amino acids in right. The five disulfide bridges are highlighted in dark grey. Two regions highlighted in light grey represent the GS domain and the L45 loop structural motif. (**b**) Protein sequence showing the preserved pattern in leech ALK4/5. Sequence alignment with human TGF-β type I receptor (ALK5, P36897) and Activin type I receptor (ALK4, P36896). High and low consensus homologies are represented by red and blue residues, respectively. (**c**–**g**) Fluorescence *in situ* hybridization on whole mounted leech CNS 24 h after lesion. Confocal microscopy images show mRNA localization using an antisense *alk4/5* riboprobe (green) in the ganglion (**c**) and in the connective (**e**) as framed in the CNS diagrams. (**f**) Arrows show more closely the *alk4/5* mRNA positive microglial cells in the point of injury. (**d**,**g**) No signal was detected with sense probes used as negative controls. Microglia cell nuclei were stained with Hoechst 33342 (blue).

Several features allowed us to identify this leech receptor as an Activin receptor-Like Kinase 4/5 (ALK4/5) orthologue (Fig. 2a). First, while disulfide bridges were common to TGF-β types I and II receptors (including Cys28-Cys49, Cys43-Cys66, Cys81-Cys95 and Cys96-Cys102), a specific disulfide bond was only present in TGF-β type I (Cys30-Cys36)^28^. Second, a type I receptors presented a GS domain possessing a SGSGSG sequence embedded in a 30-amino acid long motif. Studies show that this domain is phosphorylated by the type II receptor to play a role in receptor-mediated signaling^29,30^. Third, the leech protein showed the ADNKDNGTW amino acid sequence. This motif was described in a specific L45 loop in type I receptors (ALK4 and ALK5) necessary to activate the Smad 2/3 pathway (Fig. 2b)^31,32^.

In the lesioned nerve cord (24 hours post-lesion), a high level of *alk4/5* mRNA was detected in neuronal cell bodies located in ganglia (Fig. 2c) and in some microglia recruited to the lesion (Fig. 2e,f). The analysis of different focal planes suggested that only a small percentage of the microglia population (detected by a nuclear dye) expressed *alk4/5* gene. In both cases the negative control using a sense probe showed no signal (Fig. 2d,g).

### ALK4/5 is involved in microglia chemotaxis

**Figure 3:**
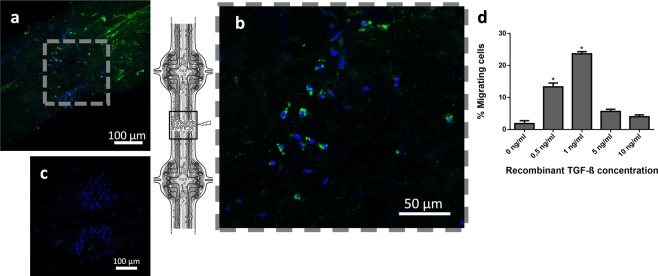
ALK4/5 in the recruitment of microglial cells at point of lesion. (**a**–**c**) Confocal microscopy analysis of ALK4/5 immunofluorescence using rabbit anti-ALK5 antibodies. (**a**) Detail of stained injured connective (site of lesion) 6 h after lesion. (**b**) Magnification of frame (**a**) showing microglial cells staining observed in the point of lesion (as framed in the CNS diagram). (**c**) No signal was detected in connective treated only with secondary antibody as negative control. Cell nuclei were stained with Hoechst 33342 (blue). (**d**) Chemotactic effect of human TGF-β on leech microglia migration. Chemotaxis assay was made with recombinant TGF-β gradient (0, 0.5, 1, 5, 10 ng/mL).

The identification of ALK4/5 expression confirmed the presence of an ALK4/5^+^ microglia subpopulation among the whole microglia population recruited to the injury site (Fig. 3a,b). Indeed, ALK4/5-negative microglia were observed in the lesion site within 6 hours post-lesion, suggesting that additional chemotactic signals were involved (Fig. 3b). A negative control using secondary antibody showed no immunopositive signal (Fig. 3c).

To study the possible involvement of this receptor in the microglia recruitment, *in vitro* chemotaxis assays were performed to measure the reactivity of freshly isolated microglia to a gradient of recombinant form of human TGF-β1, the natural ligand for ALK4/5^+^ microglia. Microglial cells were counted before cell deposition and after their migration to the arrival well. The results showed a dose-dependent chemotactic effect, resulting in a typically bell-shaped curve (Fig. 3d). Indeed, the results significantly showed that about 24% of microglia cells were reactive to the human TGF-β1 cytokine in an optimal concentration (1 ng/mL). Taken together, the results suggest that the ALK4/5^+^ microglia represent a subset of total collected microglia.

### nGDF, a neuronal orthologue of GDF8/11, is fully involved in ALK4/5-dependent microglia recruitment to the lesion

**Figure 4:**
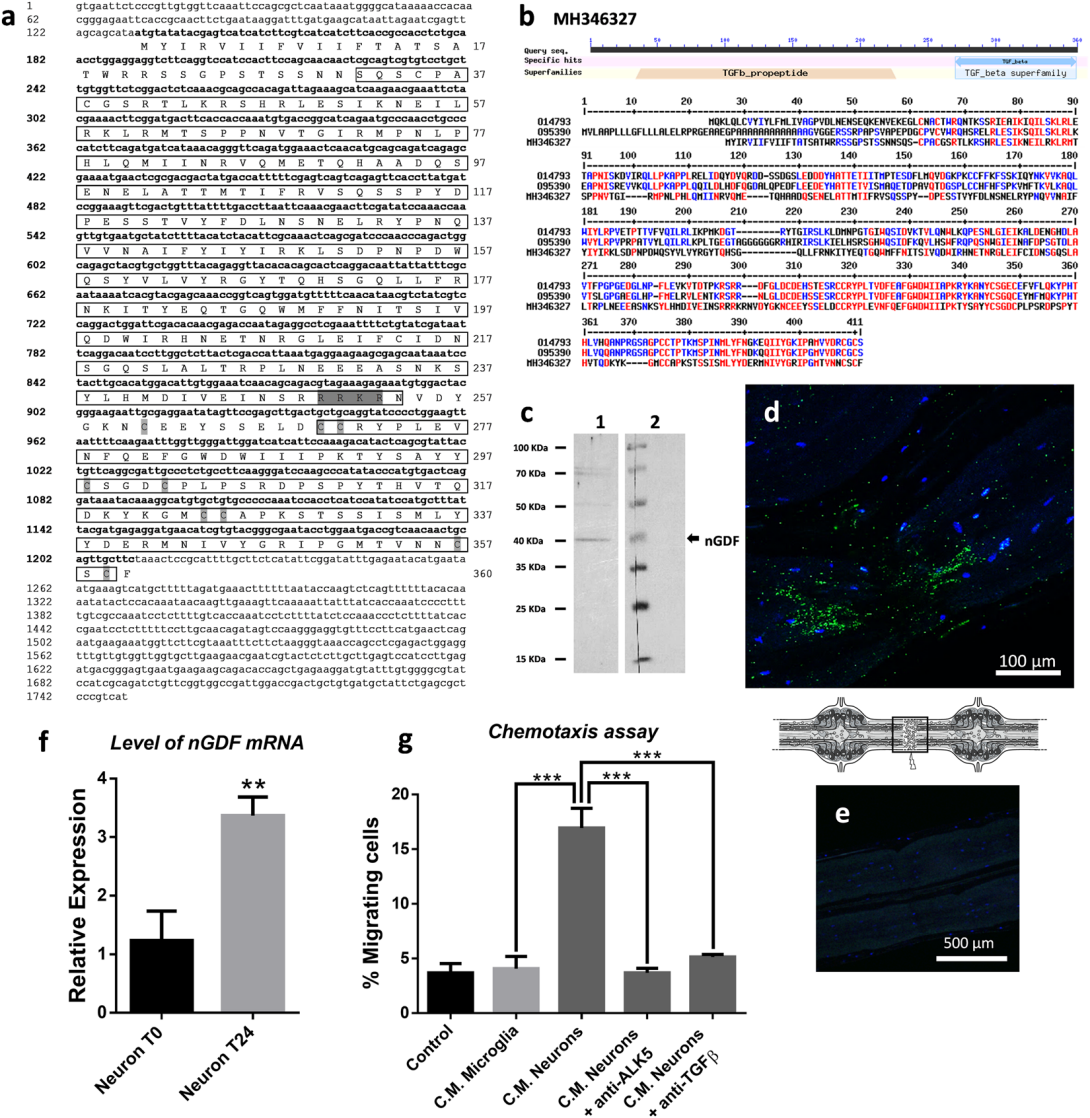
Molecular characterization of nGDF (TGF-β family member) in leech. (**a**) Nucleotide and amino acid sequences of leech nGDF. The numbers of nucleotides are indicated in left and of amino acids in right. The protein sequence of nGDF presents a propeptide and a mature form region, both framed. The nGDF protein sequence contains RRKR cut site and nine conserved cysteine residues, highlighted in dark grey and light grey, respectively. (**b**) Protein sequence showing the preserved pattern of TGF-β family in leech nGDF and sequence alignment with human GDF8 (O14793) and GDF11 (O95390). High and low consensus homologies are represented by red and blue residues, respectively. (**c**) Western blotting analysis from CNS protein extract using polyclonal rabbit anti-TGF-β antibodies (lane 1) compared to secondary antibody alone (lane 2) as a negative control (see also Supplementary Fig. S3 to have the overview of both membranes). (**d**) Immunofluorescence using polyclonal rabbit anti-TGF-β antibodies of nGDF in the point of lesion 15 minutes after-lesion, as framed in the CNS diagram. (**e**) No signal was detected in connective treated only with secondary antibody as negative control. Cell nuclei were stained with Hoechst 33342 (blue). (**f**) Real time quantitative RT-PCR of the *ngdf* mRNA level in neurons from T0 (15 min post-lesion, black) vs. 24 h post-lesion (gray) CNS. A leech 18 S ribosomal RNA was used as internal reference. Significance (*p < 0.05, **p < 0.01 vs. T0) was calculated by paired T-test (bar represents Standard Errors of Mean). (**g**) Chemotactic effect on microglia cells of conditioned medium (C.M.) from primary culture of microglia (light gray) or neurons (dark gray). The chemotactic effect of C.M. from neurons was also assessed on anti-ALK5-incubated microglia to specifically neutralize ALK4/5 in microglia (dark gray). The chemotactic effect of anti-TGF-β-incubated C.M., specifically neutralizing neurons-derived nGDF, was also evaluated on microglia (dark gray). A control medium was used as negative control (black). Significance (*p < 0.05, **p < 0.01, ***p < 0.001) was calculated by ANOVA Dunnett’s multiple comparisons test (bar represents Standard Errors of Mean).

We investigated the existence of TGF-β family members corresponding to ALK4/5 ligands. The use of the human TGF-β1 (known as natural ALK5 ligand) sequence in a local BLAST of leech databases allowed the detection of a GDF8/11-like sequence (Fig. 4a). Indeed, following a comparison with all human TGF-β family members, the leech amino acid sequence presented typical TGF-β conserved domains (Fig. 4b). It also presented 32% identity and 53% homology with human GDF8 precursor (*alias* myostatin) and 34% identity and 49% homology with human GDF11 precursor. The active peptide region was even more homologous to both molecules (60% and 62% to GDF8 and GDF11, respectively). GDF8 and GDF11 were described to predominantly use ALK4 or ALK5 type I receptors in close interaction with type II receptors (Activin receptor kinase II-A and II-B)^33^. In addition, we detected the presence of this unique leech protein band in immunoblot analysis from nerve cord protein extracts (Fig. 4c). No other molecule was detected in the nerve cord. By taking into account these sequence similarities and the existence of the ALK4/5 receptor in the leech nerve cord, the leech TGF-β member was named nGDF (for nervous Growth and Differentiation Factor).

The *in vivo* immunofluorescence analyses showed a fast accumulation of nGDF close to the injured axons only 15 minutes post-lesion (Fig. 4d) before any recruitment of microglia. No signal was observed using only secondary antibody as negative control (Fig. 4e). Importantly, *ngdf* mRNA was significantly up-regulated in primary neurons after a 24 hour culture (Fig. 4f). In addition, freshly dissociated neurons and microglia were separately cultured for 15 minutes in order to collect their respective conditioned-medium (C.M). The chemotaxis assays (performed to measure the reactivity of freshly isolated microglia) showed that C.M. from neurons presented a potent chemotactic function compared to that from microglia or control medium (Fig. 4g). Interestingly, this neuron-dependent chemotaxis was significantly reduced when target microglia were incubated with anti-ALK5 antibody. In addition, after validating the specificity of the anti-human TGF-β1 antibody to the leech nGDF, this antibody was used in *in vitro* chemotaxis assays to neutralize nGDF in the neuron-conditioned medium. In this condition, the result showed that the recruitment of microglial cells was inhibited as similarly observed in the condition using the anti-human ALK5 antibody (Fig. 4g). Taken together, the results show the neuronal origin of nGDF and its chemotactic property directed to ALK4/5^+^ microglia. Its release from the lesioned axons within the first minutes following the lesion also suggests its high importance in microglia chemoattraction. That is why, the importance of nGDF was evaluated in the time-course of microglia recruitment following injury.

### ALK4/5-dependent microglia contribute to the early phase of microglia recruitment

**Figure 5:**
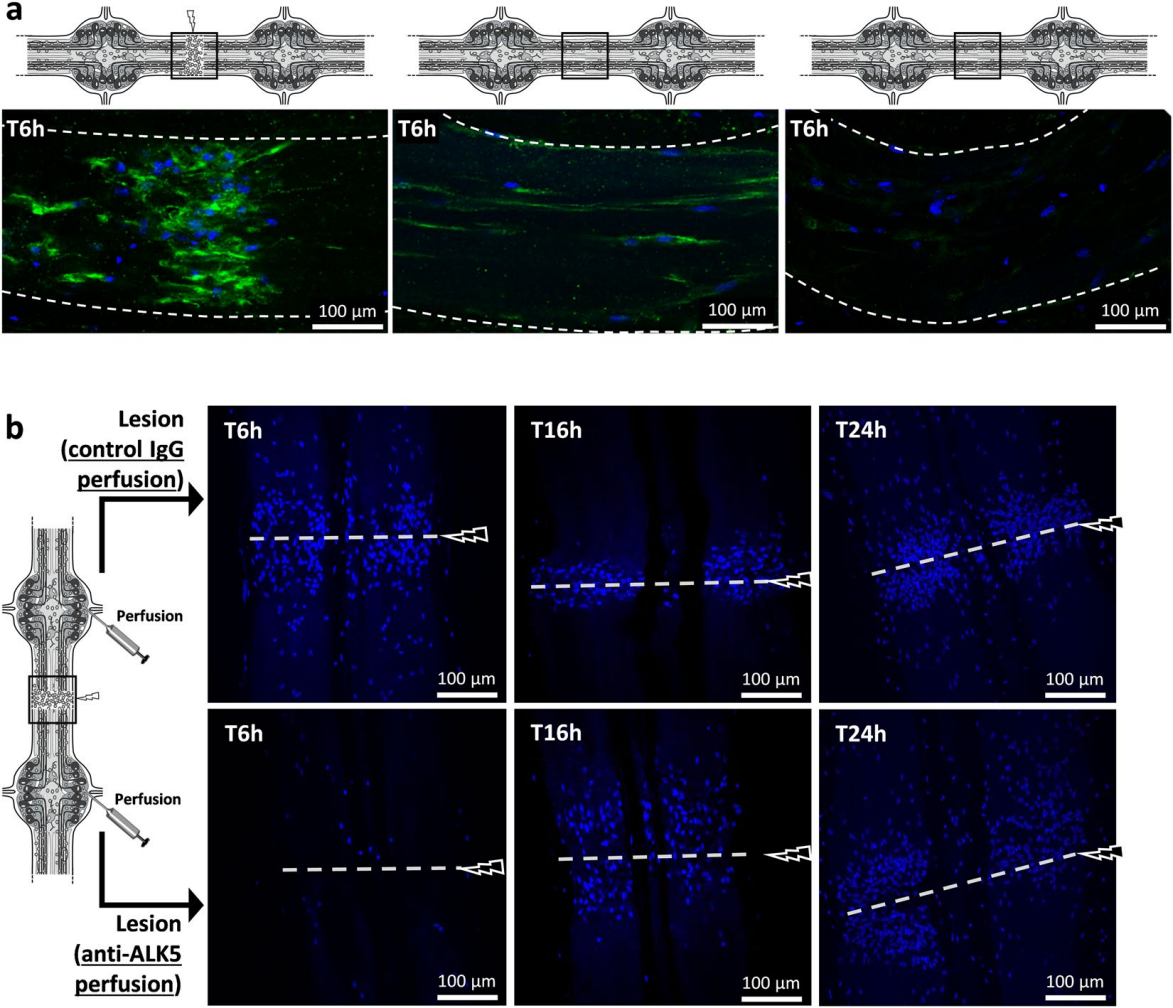
*Ex vivo* ALK4/5-dependent microglia recruitment assays. (**a**) Confocal microscopy analysis of Iba1 immunofluorescence using rabbit polyclonal anti-Iba1 antibodies 6 h after a lesion (left) or 6 h after no lesion (middle and right). The location of each image is framed in each diagram. The right image corresponds to a negative control using the secondary antibody alone. (**b**) Confocal microscopy analysis of the injured connectives (framed in the diagram) 6 h vs. 16 h and vs. 24 h after lesion. Microglia recruitment was followed by using a fluorescent nuclear dye Hoechst 33342 (blue) because only microglia are able to migrate towards the lesion. As shown in the diagram, before the lesion, rabbit polyclonal anti-ALK5-perfused connectives (below) were compared to rabbit control IgG-perfused connectives (above).

In the leech CNS, the microglia are the only cell population able to migrate towards the injury^19^. They were stained and followed using the Iba1 marker (Fig. 5a) as previously described^8^. In the injury site, 6 hours after the lesion, the results showed that only microglia accumulate in the point of the lesion. Therefore, they were studied *ex vivo* throughout their accumulation using only a fluorescent nuclear dye, allowing to avoid any detection of neutralizing antibodies after their perfusion. The lesion site was observed at three different times post-lesion by using perfusions of either anti-ALK5 antibody, to neutralize ALK4/5^+^ microglia, or rabbit IgG as negative control (Fig. 5b). The images, representative of independent triplicates, showed that the ALK4/5 neutralization affected microglia recruitment to the lesion within 6 hours post-injury compared to similar timing with Ig control perfusion. In a later time point (T16h post-lesion), the microglia accumulation was not inhibited but delayed in the migration process after the ALK4/5 neutralization, suggesting that other chemotactic signals could be involved at this time point. Finally, the comparison of both perfusions in T24h post-lesion showed no significant differences, though anti-ALK5 was still perfused in a long term. Taken together the results show that ALK4/5 pathway participated to the early phase of microglia recruitment to the lesion but is no longer used to recruit microglia in the following hours.

### Lesion-specific protein signatures in time course- and ALK4/5-dependent manner

**Figure 6:**
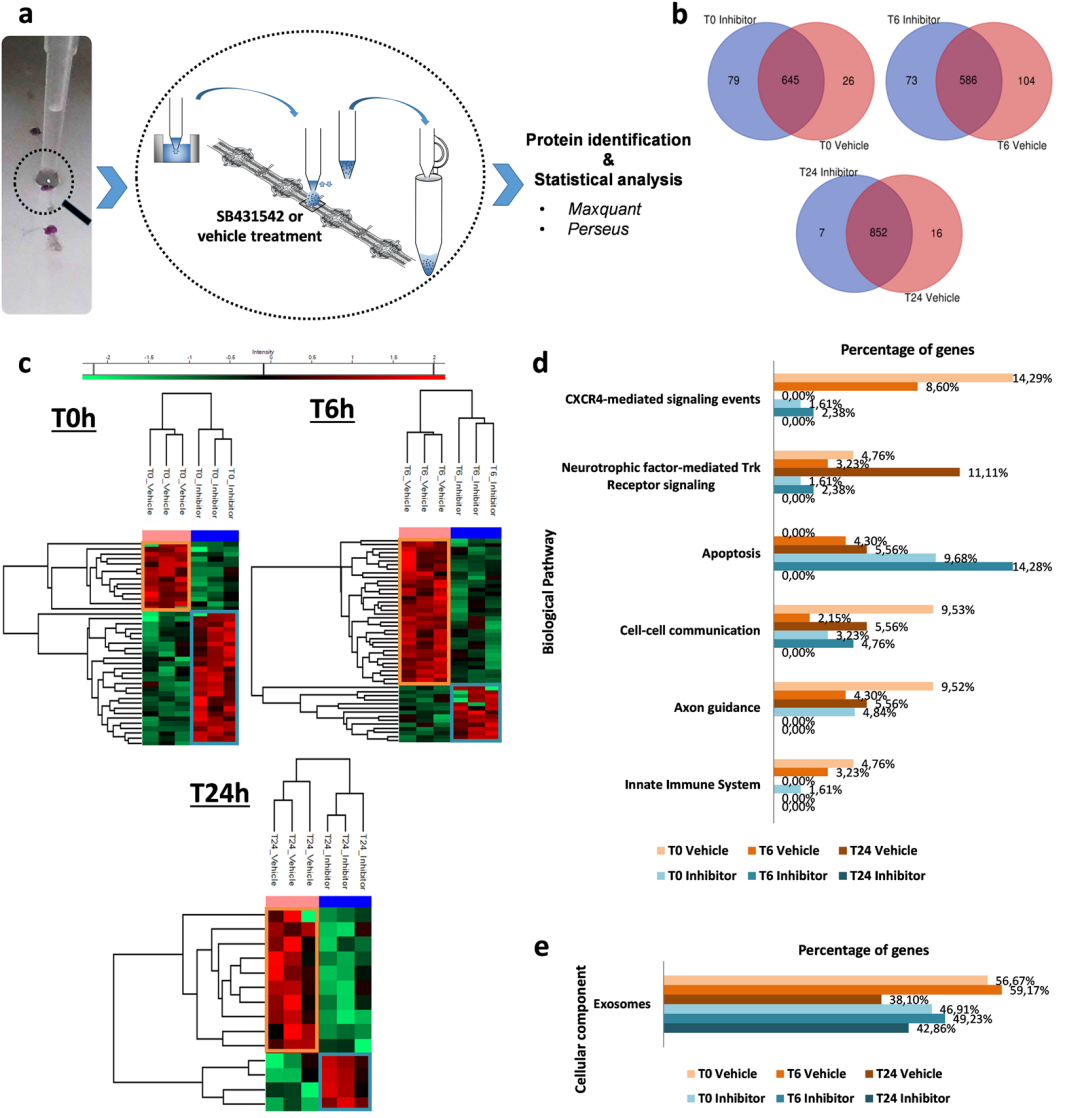
Time-course analysis of protein signatures in SB431542- vs. vehicle-conditions. (**a**) Representative image of the LESA procedure at the three points of lesioned nerve cord (in purple) followed by LC-MS/MS analysis. This procedure was performed in injured nerve cords either in SB431542 inhibitor or vehicle incubation as control. (**b**) Venn Diagrams of the numerical values for common and exclusive proteins comparing SB431542 and vehicle conditions at T0, T6h or T24h. (**c**) MaxQuant and Perseus softwares generate heatmaps of common proteins showing clusters of significantly overexpressed proteins in vehicle (highlighted in orange) and inhibitor conditions (highlighted in blue). (**d**) Analysis of biological pathway Gene Ontology (GO) with exclusive (Venn diagram) and overexpressed proteins (heatmap). The graph shows the percentage of proteins identified into designated GO categories, relative to the total number of proteins, comparing vehicle (shades of orange) to SB431542 conditions (shades of blue). (**e**) The graph shows the percentage of proteins identified into GO category Exosomes, relative to the total number of proteins. See also Supplementary Fig. S1.

Because the results showed the involvement of the ALK4/5 pathway in the crosstalk between microglia and neurons, the protein profiles throughout the microglia recruitment were studied in the lesion site using a Liquid Extraction Surface Analysis (LESA) approach. This spatially- and temporally-resolved proteomic study was performed in the lesion site from isolated fragments of nerve chain respecting the integrity of several ganglia joined by connective tissues (Fig. 6a). The leech nerve cord was injured by cutting one of the two connectives between each pair of ganglia (3 extraction points per fragment). These injured tissues were incubated with a specific inhibitor of ALK4/5 pathway (SB431542) or with its vehicle (100 mM DMSO)^34^.

Whatever the time and/or conditions, the results identified condition-exclusive protein signatures (Supplementary Fig. S1a) that were listed in Supplementary Table S1. In order to get accurate information about the molecular processes in the lesion site, the common protein signatures were analyzed to only represent those presenting a relative abundance between conditions. The results showed well discriminated clusters of over-represented protein signatures in time-dependent profiles (T0 vs T6h vs T24h) with or without SB431542 (Supplementary Fig. S1b; Supplementary Table S2). The presence of SB431542 led to a relevant decrease in the number of TGF-β signaling-associated proteins in response to the lesion (Supplementary Fig. S1c). In order to highlight the most important molecular events in the lesion site, we investigated the protein signatures by comparing SB431542- vs. vehicle-conditions for each time independently. The results showed exclusive (Fig. 6b) and over-represented (Fig. 6c) protein signatures in each condition. The exclusive protein signatures (SB431542 vs. vehicle) showed more displays in T0 (79 and 26) and T6h (73 and 104) compared to T24h (7 and 16) post-lesion conditions (Fig. 6b). In addition, regarding the common signatures, their analysis showed clusters containing a higher number of over-represented proteins in T0 and T6h compared to T24h post-lesion (Fig. 6c). From exclusive proteins (Supplementary Table S3) and over-represented proteins (Supplementary Table S4), a first analysis consisted in identifying proteins that were specifically detected in only one time post-lesion (represented in bold in Tables S3 and S4). Then, in each time post-lesion, the protein signatures were compared between SB431542- and vehicle-conditions in order to highlight the molecular events which could be the consequences of an ALK4/5 inhibition. Some proteins exclusively or over-represented in T0 following the ALK4/5 inhibition were already described to be involved in neurodegenerative diseases. They included for example the succinate-CoA ligase (SUCLG1), the NADH dehydrogenase complex (NDUFA10, NDUFS2), the cytochrome c (CYCS), the oxoglutarate dehydrogenase-like protein (OGDHL), the ATP synthase subunits (ATP5J2, ATP5A1) and the phosphoglycerate kinase 2 (PGK2).

In addition, the exclusive proteins (Supplementary Table S3) were merged to over-represented proteins (Supplementary Table S4) in a global analysis in order to compare functional processes involved throughout the response to the lesion. The most affected biological pathways by ALK4/5 inhibition were associated to (i) CXCR4-mediated signaling events, regulating synaptic function and neuronal survival^35,36^; (ii) Neurotrophic factor-mediated Trk receptor signaling, leading to axon regeneration^37^; (iii) Apoptosis; (iv) Cell-cell communication; (v) Axon guidance and (vi) Innate immune reactions (Fig. 6d). The related proteins were detailed in the Tables S3 and S4. These functional mechanisms were down-regulated in the lesion site following ALK4/5 inhibition, excepted for the apoptotic functions. It demonstrated that the ALK4/5 pathway was a key process leading to axon regeneration. Interestingly, when we considered only the vehicle conditions, the comparison between T0/T6h and T24h post-lesion showed the early involvement (T0/T6h) of CXCR4-mediated signaling whereas the neurotrophic factor-mediated Trk receptor signaling was mainly detected as a later mechanism (T24h). These results highlighted a new hypothesis about the functional time-program of recruited microglia subpopulations. The components associated to exosomes were also an important signature. Indeed, they were highly represented in the response to lesion (Fig. 6e; Supplementary Table S5). As represented in T6h vehicle condition, they represented up to 59% of detected protein signatures (Supplementary Fig. S1d). While the importance of the ALK4/5 pathway were demonstrated in the early phase of microglia recruitment to lesion, the wide time-course proteomic analysis also showed that the ALK4/5 inhibition can modulate early (T0 and T6h) as well as late (T24h post-lesion) biological processes. This logically raised the question of the role of ALK4/5 in the subsequent later response, which prompted us to consider its importance until T24h post-injury. The results suggest that ALK4/5 signaling contributes to a time-program in the nerve repair.

### ALK4/5-dependent neurons mediate a late phase of microglia recruitment

**Figure 7:**
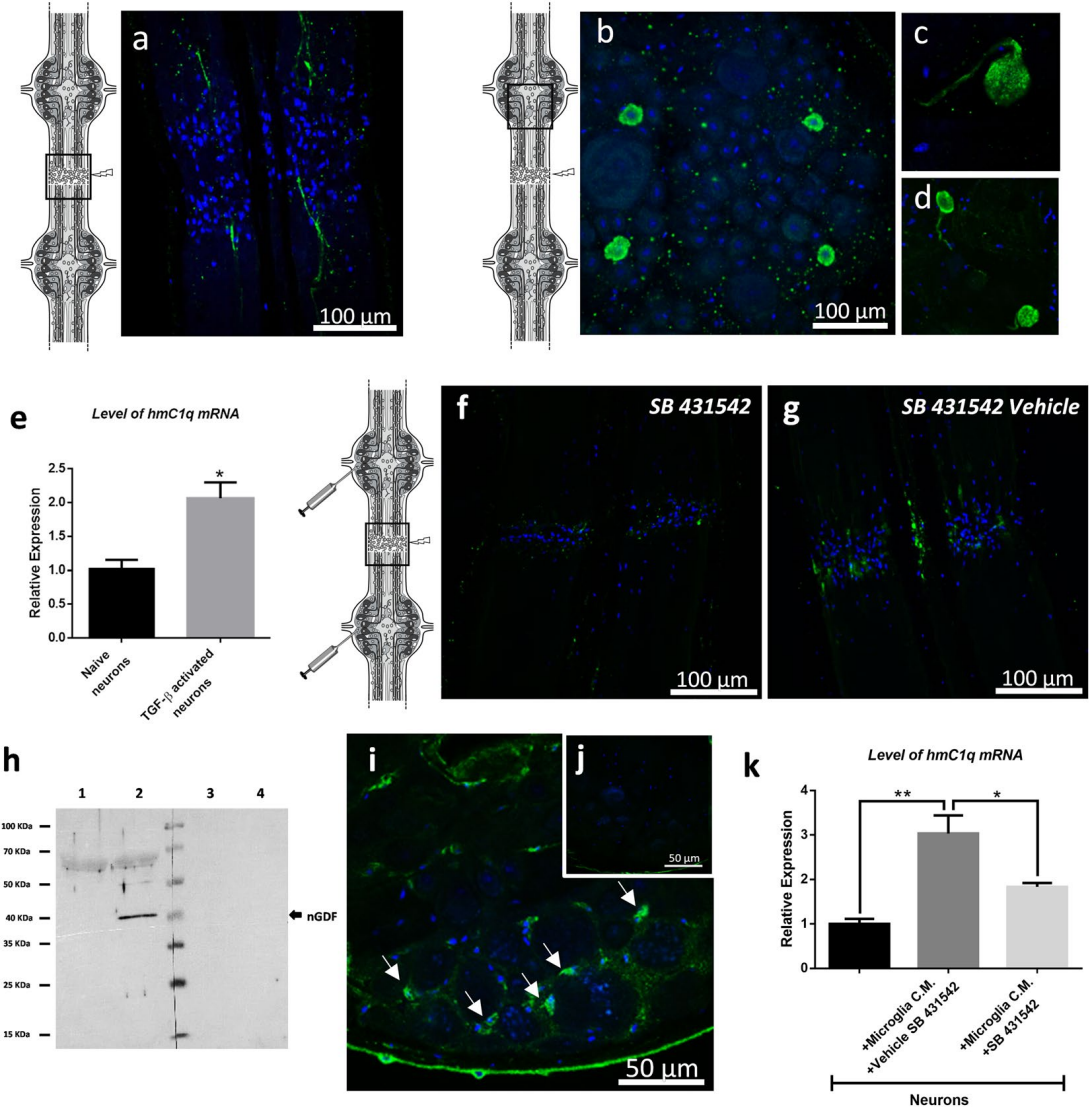
ALK4/5-dependent neurons mediate the C1qBP+ microglia cells accumulation. (**a**–**d**) Confocal microscopy analysis of ALK4/5 immunofluorescence in lesioned nerve cord post-injury using rabbit polyclonal anti-ALK5 antibodies. (**a**) Injured axons are stained in connective 16 h after lesion. (**b**–**d**) Some neuronal cells bodies are stained in ganglia showing the proximal end of axons (**c**,**d**). (**e**) Real time quantitative RT-PCR of *hmc1q* mRNA level in naive vs. TGF-β-activated neurons. A leech 18 S ribosomal RNA was used as internal reference. Significance (*p < 0.05 vs naive neurons) was calculated by paired T-test (bar represents Standard Errors of Mean). (**f**,**g**) Confocal microscopy analysis of C1qBP immunofluorescence using rabbit polyclonal anti-C1qBP antibodies in lesioned nerve cord 24 h post-injury. As shown in the diagram, SB431542 inhibitor-injected nerve cords (F) were compared to vehicle-injected nerve cords (**g**) as negative control. Microglia cell nuclei were followed using Hoechst 33342 (blue). (**h**) Western blotting analysis from neuron (lanes 1,3) and microglia (lanes 2,4) protein extracts collected from lesioned nerve cord (24 h post-injury) using rabbit polyclonal anti-TGF-β antibodies (lanes 1,2) compared to secondary antibody alone as a negative control (lanes 3,4) (see also Supplementary Fig. S3 to have the overview of both membranes). (**i**,**j**) Fluorescence *in situ* hybridization on whole mounted leech CNS 24 h after lesion. Confocal microscopy images show *ngdf* mRNA localization using an antisense riboprobe (green) in ganglionic microglia (**i**, arrows) and no signal using sense riboprobe as negative control (**j**). (**k**) Real time quantitative RT-PCR of the *hmc1q* mRNA level in naïve vs. microglial conditioned medium (CM)-activated neurons. The CM-activated neurons was incubated with either SB431542 inhibitor or vehicle. A leech 18S ribosomal RNA was used as internal reference. Significance (*p < 0.05, **p < 0.01) was calculated by ANOVA paired T-test (bar represents Standard Errors of Mean). See also Supplementary Fig. S2.

The analysis of the ALK4/5 distribution was performed in the connective tissues and demonstrated that the receptor was present in a few injured axons 16 hours post-injury (Fig. 7a). At this time, ALK4/5 was no longer detectable in the accumulated microglia. This result confirmed that the ALK4/5-dependent microglia recruitment only occurred in early hours post-lesion (Fig. 5). In ganglia, the results showed an ALK4/5 signal in only a few symmetric neurons in each ganglion 16 and 24 hours post-injury (Fig. 7b). A stacking of focal plans in the ganglion also revealed the ALK4/5-immunopositive signal in the axons (Fig. 7c,d) as also observed near the lesion in the connectives (Fig. 7a).

Because we showed that the ALK4/5 inhibition led to significant changes in the connective tissue until 24 hours after the lesion, we decided to study the importance of these ALK4/5^+^ neurons in the ganglia, especially their contribution to other chemotactic signals allowing the microglia recruitment.

During the neurogenesis in mammals, the neuronal reactivity through TGF-β receptors induces the neuronal production of C1q and C3 complement factors involved in a microglia recruitment allowing the synaptic pruning^27,38^. Since neuronal *Hm*C1q was described in the leech microglia recruitment^13,14,16^, we investigated a possible correlation between the ALK4/5 pathway and C1q production in neurons. The neuronal reactivity was studied *in vitro* by the exposure of primary neurons to a recombinant form of TGF-β1 (ALK5 natural ligand) during 24 hours. The results showed that *hmc1q* mRNA was significantly induced in TGF-β-activated neurons compared to naïve ones (Fig. 7e). Thus, the ALK4/5^+^ neurons were involved in the production of neuronal C1q that we previously described as one chemotactic signal for C1qBP-dependent microglia accumulation to the lesion site^13,14^. In order to assess the importance of the ALK4/5 signaling in the *Hm*C1q-activated microglia recruitment, C1qBP^+^ microglia cells were followed in lesioned connectives 24 hours post-lesion under an ALK4/5-specific inhibitor (SB431542) (Fig. 7f,g). Because ALK4/5^+^ microglia were no longer involved in the late phase of the recruitment to the lesion (Fig. 5), the SB431542 inhibitor was directed to ALK4/5^+^ neurons only. The results demonstrated that a SB431542 perfusion specifically reduced the number of the C1qBP^+^ microglia cells recruited to the lesion (Fig. 7f) compared to a similar tissue perfused with the SB431542 vehicle (Fig. 7g). Taken together, these results demonstrate that ALK4/5^+^ neurons contribute to the accumulation of C1qBP^+^ microglial cells in the later phase of the recruitment to the lesion, by stimulating a neuronal *Hm*C1q production. These ALK4/5^+^ neurons could directly produce *Hm*C1q as a chemotactic factor and/or even propagate a larger signal towards other neurons contributing to a wider release of *Hm*C1q in axons at the lesion site.

### Activated ganglionic microglia also produce nGDF to influence neurons

The only ligand for TGF-β type I receptor that we detected in databases was nGDF. Since ALK4/5^+^ neuronal cell bodies were involved 24 hours post-lesion in the induction of *Hm*C1q (Fig. 7e–g), protein extracts from neurons and microglia were analyzed for nGDF signature (Fig. 7h). The results showed at 24 hours post-lesion the presence of this factor in microglia but not in neurons. This result was corroborated by a specific *ngdf* mRNA location in ganglionic microglia close to neuronal cell bodies (Fig. 7i). A negative control using sense riboprobe showed no signal (Fig. 7j). The ability of microglia to induce a neuronal *Hm*C1q production was confirmed using primary microglia and neurons in co-culture experiments. The level of *hmc1q* mRNA in primarily cultured neurons was significantly induced in the presence of microglia compared to a mono-culture of primary neurons (Fig. 7k). The induction of *hmc1q* mRNA level in neurons was partially but significantly inhibited when SB431542 inhibitor was added to the co-culture media. This demonstrated that some microglia cells regulated a neuronal *Hm*C1q production through the ALK4/5 pathway. This inhibitor clearly targeted the neuronal ALK4/5 since no microglia in ganglia were able to expose this receptor at any time. The immunofluorescence analyses in the nerve cord also confirmed the nGDF protein location in ganglionic microglia cells, in similar regions to those observed for *ngdf* mRNA, as well as in interneuronal spaces (Supplementary Fig. S2a) compared to negative control (Supplementary Fig. S2b). Consequently, the results suggest that some microglia cells release nGDF to interact with neuronal cell bodies. These resident microglia cells in ganglia might be functionally orientated to dialog with neuronal cell bodies. It is important to understand how they receive an activation signal to induce their nGDF production in this process. Interestingly, other immunofluorescence analyses in the ganglia revealed that the ganglionic microglia cells did not produce any nGDF when the lesion site was isolated from the ganglia by ligatures (Supplementary Fig. S2c). This suggests that factors released from damaged axons and/or microglia in the connectives could distantly activate ganglionic microglia to interact with corresponding neuronal cell bodies.

## Discussion

Microglia are motile sensor cells significantly contributing to the nervous system development. It is crucial to investigate their functions at the crossroads of neuronal and immune pathways. Dysfunctional microglia are more and more correlated to a high level of neuroinflammation leading to neuropathologies^4,39^. Thus, understanding and controlling microglia activities could bring a novel therapeutic way.

In mammals, the cell diversity and plasticity require discrimination between resident microglia and meningeal as well as plexus choroid macrophages. New microglia-specific markers are investigated, notably revealing the involvement of TGF-β receptors, purinergic receptor P2RY12, scavenger receptor FCRLS and transmembrane protein TMEM119^23^. TGF-β receptors are demonstrated as crucial in the regulation of the microglial maturation. A similar observation is performed between microglia and peritoneal macrophages demonstrating a preferential expression of mRNAs encoding TGF-β receptors *tgfbr1* (alias *alk5*) and *tgfbr2* in microglia^22^. TGF-β ligands are described to exert a privileged effect on the microglia-specific program of gene expression compared to a M-CSF (Macrophage colony-stimulating factor) influence^22^. Finally, this TGF-β receptor exposure is specified as having an influence on microglia-specific transcription factor Sall1 to maintain a physiological state of the adult microglia^25^. Understanding the use of the TGF-β signaling is hence crucial to specify the physiological functions of microglia. This is why, we investigated the location of such TGF-β receptors in the medicinal leech CNS in order to better understand TGF-β signaling functions.

As described in vertebrates, the leech microglia are also motile cells migrating towards lesions after morphological changes^12^. Their study is even more important since we know that their accumulation to the lesion is supported by chemotactic signals^8,12–15^ and is essential to initiate a nerve repair program^20^. The tubular architecture of the leech CNS and the particular antero-posterior axonal projection of many neurons allow *in vivo* or *ex vivo* studies from isolated fragments of nerve cord. Thus, in the present report, a specific mechanical lesion was carried out in the middle of connectives to damage the axons while the neuronal cell bodies were still intact. This experimental manipulation allowed to study the processes leading to immune reactions, axonal guidance, axonal sprouting and synaptogenesis in close relation to microglia functions.

The results showed in microglia and also in neuron subsets the existence of a TGF-β type I receptor corresponding to an orthologue of ALK4 (Activin type I receptor) and/or ALK5 (TGF-β type I receptor) (Figs 2 and 3). The leech ALK4/5 possesses amino acid residues belonging to the TGF-β family type I receptor^28,29,31,32^. The present report showed the use of the ALK4/5 pathway in the microglia recruitment and the existence of a member of the TGF-β family similar to GDF8 (*alias* myostatin) and GDF11 both recognizing ALK4 or ALK5^40,41^. This molecule, named nGDF, was released by injured neurons (Fig. 4) to contribute within the first minutes to the microglia mobilization. But, nGDF was no longer used in the following hours though microglia were still recruited to the lesion (Fig. 5). Taken together, the results demonstrate the early involvement of GDF8/11 orthologue (nGDF) in the microglia recruitment to the axonal lesion. The functions supported by these microglial populations in contact with injured axons are not yet understood. In mammals, GDF11 negatively controls a neuronal proliferation through ALK4/5 type I receptors^40,41^ and *in vitro* assays suggest a comparable role of GDF8 in the brain^40^.

In order to explore this time-program throughout the response to the injury, we used a Liquid Extraction Surface Analysis (LESA) technique in the lesion site (Fig. 6 and Supplementary Fig. S1). A first comparison of time-specific proteins was performed between the ALK4/5 inhibition and control (Supplementary Tables S3 and S4). The analysis of the protein signatures after the ALK4/5 inhibition (T0) allowed the detection of over-represented hypoxia-associated proteins that are related to pro-inflammatory microglia and potentially involved in Alzheimer or Huntington diseases^42^. It included for example the succinate-CoA ligase, the NADH dehydrogenase complex, the cytochrome c, the oxoglutarate dehydrogenase-like protein, the ATP synthase subunits and the phosphoglycerate kinase 2. Thus, the protein signatures following the ALK4/5 inhibition suggested a switch to a neurodegenerative disease profile. Twenty-four hours after the ALK4/5 inhibition, only 3 specific proteins were over-represented (SSUH2, TPM2, PPP3CB). In contrast, FLNC, HSPA8 and NTRK2 proteins were stimulated in the control. Because NTRK2 is a natural receptor for neuronal growth factors (BDNF and NTF4), its over-representation in the control suggested the involvement of the ALK4/5 pathway in a neurite outgrowth process^43,44^. The other analyses involving the detection of biological processes showed the early identification of CXCR4-mediated signaling pathway, really important in T0 and T6h conditions but absent in T24h post-lesion. In mammals, CXCR4 is known to be expressed by microglia to regulate microglial colonization under CXCL12 influence^45^. This activation is also described as an important signature of the early stage in embryonic microglia^46^. Finally, the CXCR4 signaling could first protect neurons from cell death which is typical of early events after injury prior to later engage sprouting and synaptogenesis mechanisms^35^. The results showed that this CXCR4 pathway was remarkably downregulated after the ALK4/5 inhibition and revealed its involvement in the early response. Thus, the first hours of the response to the lesion involved a protected environment facilitated by the recruitment of early stage-specific microglia. Interestingly, other biological pathways were significantly increased in the later response to lesion (T24h post-lesion) compared to the earlier ones (T0 and T6h). It included especially the neurotrophic factor-mediated Trk receptor signaling resulting in growth and axon regeneration following injury^37^. This time-program developing chronological functions throughout the nerve repair was also strongly impaired when ALK4/5 was inhibited as revealed by the significant decrease of axon guidance processes. Conversely, the proteomic analysis of the lesion showed that the ALK4/5 inhibition induced apoptotic events suggesting a cell death after injury. Thus, the ALK4/5 pathway is necessary in this time-dependent process. The detection of differential protein signatures after the ALK4/5 inhibition was expected in the early hours post-lesion. Nevertheless, this study also reveals the longer term importance of the ALK4/5 pathway in the response to lesion (T24h post-lesion).

Another remarkable functional process was demonstrated by the detection of proteins involved in the production and accumulation of Extracellular Vesicles (EVs) such as exosomes and microvesicles to the lesion (Fig. 6 and Supplementary Fig. S1). The relative abundance of these proteins suggested that EVs were released by neurons and/or recruited microglia throughout the response to the injury. Even after the ALK4/5 inhibition, it was possible to observe this EV-derived signature. Consequently, it suggests the EV response to be a privileged mechanism in the crosstalk between microglia and neurons in the leech. As recently described, we contribute to understand the importance of EVs in this crosstalk^47–51^.

**Figure 8:**
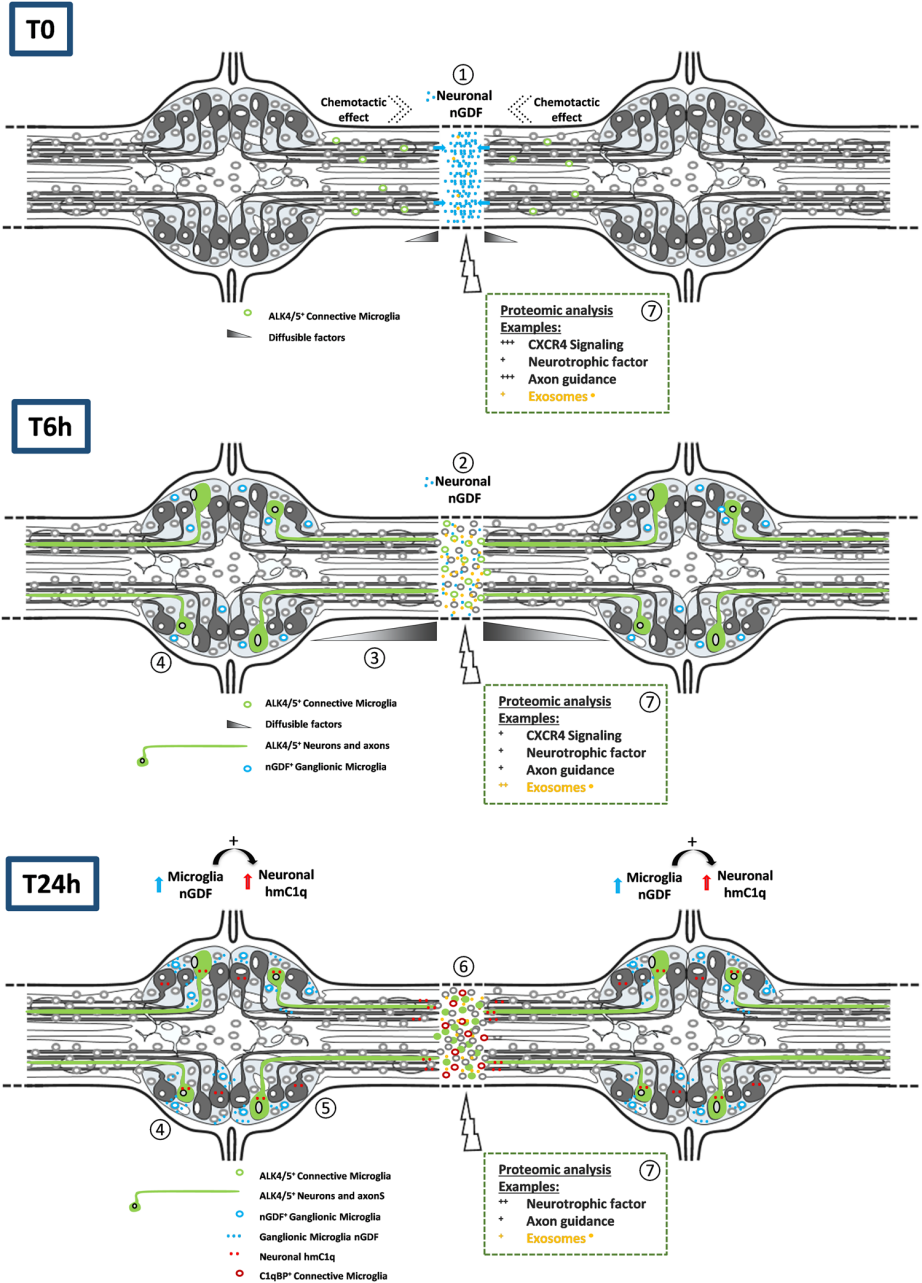
Diagram summarizing the chronological processes after axonal lesion. The ALK4/5-dependent events were described according to three times post-lesion. The early release of neuronal nGDF at lesion (1) allows the recruitment of an ALK4/5^+^ microglia subpopulation (2). Diffusible factors at lesion would distantly inform ganglionic microglia and/or neuronal cell bodies to organize the long term response (3). Some neurons expose ALK4/5 receptor and ganglionic microglia produce nGDF (4). Then, nGDF from ganglionic microglia induces ALK4/5^+^ neurons contributing to a neuronal *Hm*C1q production (5). Then a C1qBP^+^ microglia subpopulation will be recruited at lesion under *Hm*C1q influence (6). The involvement of exosomes derived from microglia and/or neurons is supposed to be remarkably important as well as other chronological signaling pathways (7).

The second part of this study showed that ALK4/5 receptor was still expressed in the lesion site 24h post-injury in a few damaged axons and also in ganglionic ALK4/5^+^ neuronal cell bodies, while microglia cells no longer used it (Fig. 7). Previous studies in mammals demonstrate the neuronal exposure of such receptors as essential in differentiation and survival^52–54^. The neuronal ALK5 exposure is interestingly described as a regulator of the late stages in adult hippocampal neurogenesis^26^ and in protecting neurons from degeneration and cell death in Alzheimer pathogenesis^55–57^. However, the pathways by which these neurons could be regulated remained to be clarified because the sources of TGF-β family ligands could be multiple during the development but also the aging of the nervous system. Particularly, astrocytic TGF-β family members could activate ALK5^+^ neurons in order to produce complement factors during the neurogenesis^27^. The subsequent release of the neuronal C1q is important because it recruits C1qBP^+^ microglia, necessary in the synaptic pruning during the CNS development^38^. Since the neuronal C1q is involved in the leech microglia recruitment^13,14,16^, a possible correlation to the ALK4/5 signaling was investigated. The results showed that neurons were reactive to microglia-derived nGDF and induced a neuronal C1q production. So far, there was no evidence that ALK4/5^+^ neurons were those able to produce *Hm*C1q. Indeed, the ALK4/5^+^ neurons could directly produce *Hm*C1q as a chemotactic factor and/or propagate a larger paracrine signal towards other neighbor neurons contributing to a wider release of *Hm*C1q. This question will be addressed in further studies. Interestingly, the C1q-dependent microglia recruitment at lesion was significantly affected by neutralization of ALK4/5 signaling. These results corroborated the late importance of ALK4/5 pathway acting as a determinant loop in the crosstalk between microglia and neurons. Interestingly, the presence of ligatures preventing any physical exchange between connectives and ganglia led to the inhibition of nGDF release in ganglia. The time-program following the injury thus involved a distant communication between both regions (Fig. 8). The lesion-dependent signal that induced ganglionic microglia to release nGDF and activate neuronal *Hm*C1q production remains to be elucidated.

In conclusion, the ALK4/5 signaling is important during mammalian neurogenesis and participates to the neuronal plasticity. It could also represent a neuroprotective event in the adult CNS. The present report used the leech CNS, as a natural model of nerve regeneration, to study either axon-microglia or neuronal cell body-microglia interactions. The results demonstrate that ALK4/5 signaling is essential throughout the response to the lesion in the crosstalk between microglia and neurons. They may give a new insight into the functions of this pathway as an important signal contributing to a correct sequential mobilization of microglia leading to an axon regeneration. Elucidating the functional impact of ALK4/5 signaling in mammalian brain could help to promote specific microglia subpopulations that, properly recruited, could improve functional repair of the CNS.

## Materials and Methods

### Leech central nervous system structure

All protocols regarding the use of leeches were carried out in strict accordance with the French legislation and European Treaty, and in compliance with the Helsinki Declaration. The adult leeches *Hirudo medicinalis* were obtained from Biopharm (Hendy, UK). After anesthesia in 10% ethanol at 4 °C for 15 min, the CNS were dissected out in a sterile Ringer solution (115 mM NaCl, 1.8 mM CaCl_2_, 4 mM KCl, 10 mM Tris maleate, pH 7.4) under a laminar flow hood. After isolation of CNS, the samples were placed in 3 successive baths of antibiotics (100 UI/mL penicillin, 100 µg/mL streptomycin and 100 µg/mL gentamycin) for 15 min and later incubated in complete medium, made of Leibovitz L-15 medium (Invitrogen, Carlsbad CA, USA) complemented with 2 mM L-glutamin, 100 UI/mL penicillin, 100 µg/mL streptomycin, 100 µg/mL gentamycin, 0.6% glucose, 10 mM Hepes and 10% Exosome-depleted FBS Media Supplement (SBI System Bioscience, Palo Alto CA, USA). *In situ* hybridization and immunohistochemical analyses were performed on injured CNS by crushing the two connectives between the second and third ganglia, from a four ganglia long fragment.

### Neurons and microglial cell preparation

The whole CNS was placed in 35 mm Petri dishes with 200 µL of complete medium. Each ganglion was carefully decapsulated by removing the collagen layer enveloping the nerve cords. The nerve cells, neurons (10–70 µm) and microglial cells (5 µm), were mechanically collected by gentle scraping and filtered through different size filters for separating the population according to size. Afterwards, the cell debris were eliminated in a 100 µm pluriStrainer filter (Dominique Dutscher, Brumath, France). Microglia were selected through a filter of 6 µm pluriStrainer and the neurons were collected in the upper part of this filter. The enriched microglial cells or neurons were centrifuged at 1,200 × g for 10 min at Room Temperature (RT). The cell pellet, corresponding to one nerve cord, was resuspended in 200 µL complete medium for the migration assays. Regarding the preparation of conditioned medium, the pellet, corresponding to 10 nerve cords, was resuspended in 500 µL complete medium (See Supplementary Method 1). The cell-free supernatant of each of the microglial cells and neurons from 10 nerve cords was used as conditioned medium (CM) in the chemotaxis experiments.

### Molecular characterization

In a Hirudinea Genomics Consortium, we contributed to create a *Hirudo medicinalis* draft genome as previously described^58^. Sequences were assembled from paired short reads using Velvet and PHRAP/CONSED algorithms^59,60^ and given to GlimmerHMM to get predicted mRNA database^61^. These predicted mRNA sequences was compared in a Local BLAST program with human TGF-β type I receptor and TGF-β1 amino acid sequences^62^. The candidate sequences was submitted to Swiss-Prot databases using BLAST in order to specify similarities in TGF-β type I receptors and TGF-β superfamily respectively. From putative partial mRNA sequences, specific primers were designed to get the natural and complete sequences by RACE-PCR from CNS total RNAs (Supplementary Methods S2 and 3). PCR products were ligated into the pGEM T-easy vector (Promega, Madison WI, USA) and cloned into JM109 cells according to the manufacturer’s instructions. Finally, products were sequenced using BigDye Terminator v3.0 polymerization kit before detection on Genetic Analyzer (Applied Biosystems, Foster City CA, USA). The assembly of 5′ and 3′ end sequences allowed characterizing the full length mRNA of *tgfbr1* and *ngdf* encoding respectively ALK4/5 (GenBank accession number MH346327) and nGDF (GenBank accession number MH346328) proteins.

### Gene expression analysis

The neurons were collected, as described above, from the CNS of 10 leeches for each experimental condition and incubated in complete medium. The total RNA extraction was performed as described in Supplementary Method S2. cDNA library was generated from 2 µg of total RNA using random primers and Superscript III Reverse Transcriptase kit (Invitrogen, Carlsbad CA, USA) in a final volume of 20 µL. cDNAs were treated with RNaseH (Promega, Madison WI, USA) to optimize the amplification reaction product. Real-time quantitative PCR (qPCR) were performed with the Platinum SYBR Green qPCR SuperMix (Invitrogen, Carlsbad CA, USA) by combining 2 µL of cDNA template, 2 µL of primer mix (10 mM) and 25 µL of Platinum SYBR Green qPCR SuperMix-UDG in a final volume of 50 µL. Specific primers were designed for the qPCR analyses, for *ngdf* gene (5′-TGCTTGTGGTTCTCGGACTC-3′, 5′-TTTCGCTCTGATCTGCTGCA-3′), *hmc1q* gene (5′-GTCTCGGGAGTGCAAGGAAT-3′, 5′-TGTATTGTTCCCGACTCGCC-3′) and for a leech 18 S ribosomal RNA (5′-GGAGGAGCGCGTTTATTAAG-3′, 5′-GGGCACACACTTGAAACATC-3′), used as normalizer. The qPCR reactions were conducted on CFX 96 Real-Time System (BioRad, Hercules CA, USA) with the following conditions: 2 min at 50 °C (1 cycle), 2 min at 95 °C (1 cycle), 30 s at 95 °C, 30 s at 58 °C and 30 s at 60 °C (39 cycles) followed by a final melting curve to control the amplified specificity. The expression level of *ngdf* gene was compared between neurons 15 minutes (T0) and 24 hours after lesion (T24 h). The expression level of *hmc1q* gene was compared, at first, between naive and stimulated neurons with recombinant human TGF-β (20 ng/mL, Sigma-Aldrich, Saint Louis MO, USA), as ALK5 ligand. In a second time, *hmc1q* gene was compared between the unstimulated neurons and the neurons in co-culture with the microglia cells, separated thanks to a Transwell® porosity 0.4 µm membrane (Corning, Corning NY, USA), with or without SB431542 (20 µM, R&D Systems, Minneapolis MN, USA), an ALK5-specific and ALK4-relative inhibitor^34^. Experiments were done on triplicate samples in different sets of cDNA template. The analysis of relative gene expression of *hmc1q* and *ngdf* was calculated using the 2^−ΔΔCt^ method^63^. Statistical analyses were performed by Paired T-test using GraphPad Prism 6.0 software. Statistical differences were considered to be significant if p-value was <0.05.

### Fluorescent *in situ* hybridization (FISH)

Nerve cords were incubated 24 hours post-lesion and fixed for one hour at 4 °C in 4% paraformaldehyde. Digoxigenin-UTP-labelled specific antisense and sense riboprobes (negative control) were generated. The riboprobes of *tgfbr1* were generated from 688–1356 nucleotides sequence mRNA (Genbank Accession Number MH346327) (size 668nt) with specific Forward (5′-AAGTGTGGAGGGGTGTATGG-3′) and Reverse (5′-CTCTTCGTGCGTTGGATCAG-3′) primers and from the 156–592 nucleotides sequence of *ngdf* (Genbank Accession Number MH346328) (size 436nt) with specific primers (5′-CATCATCTTCACCGCCACCT-3′, 5′-GTTGGGATCGCTGAGTTTGC-3′). After PCR amplification and the insertion of the product in pGEM-T easy vector system (Promega, Madison WI, USA), the RNA sequence of interest was obtained by *in vitro* transcription using DIG RNA-labeling kit according to the manufacturer’s instructions (Roche Diagnostics, Risch-Rotkreuz, Swiss).

The hybridization protocol was performed in nerve cords as listed below^64^, with minor modifications (See Supplementary Method S4). After the hybridization protocol, the nerve cords were mounted on the slide with Dako Fluorescent Mounting Medium (Agilent, Santa Clara CA, USA). Slides were kept at 4 °C in the dark until observation with a Zeiss LSM700 confocal microscope connected to a Zeiss Axiovert 200 M with an EC Plan-Neofluar 40x/1.30 numerical aperture oil immersion objective (Carl Zeiss AG, Oberkochen, Germany). Processing of the images was performed using Zen software and applied on the entire images as well as on controls. The presented pictures are representative of independent triplicates.

### Immunohistochemistry

In experiments with rabbit polyclonal anti-human TGF-β1 (1/100, ab92486, Abcam, Cambridge, UK) and rabbit polyclonal anti-human ALK5 (1/250, ab125310, Abcam, Cambridge, UK) antibodies, analyses were performed on experimentally injured nerve cords, as described above, and incubated in complete L-15 medium 15 minutes (T0), 6 hours (T6 h) or 24 hours (T24 h).

The experiment using anti-human TGF-β1 was also performed with and without ligation between ganglia 2 and 3, which were tied up with a loop of fine nylon thread to confine the lesion region.

The experiments using rabbit polyclonal anti-human C1qBP (1/500, HPA026483, Sigma-Aldrich, Saint Louis MO, USA) were performed on injured nerve cords, as described above, 24 h after incubation in complete L-15 medium and with injection of SB431542 (20 µM, R&D Systems, Minneapolis MN, USA) as ALK4/5-specific inhibitor, or its vehicle DMSO (100 mM). The injection of the inhibitor was performed in decapsulated ganglia adjacent to the lesioned connective so that it reaches the injury site. For injections, patch pipettes were pulled from borosilicate glass capillaries (outer diameter 1.5 mm, Clark GC 150 F-10) using a two-stage horizontal micropipette puller (model P-97, Sutter Instrument, Novato, CA, USA) (pipette resistance 3 to 5 MΩ).

The experiments using rabbit polyclonal anti-leech Iba1 antibodies (1/5000) were performed on intact or injured nerve cords 6 h after incubation in complete L-15 medium as previously described^8^.

After the different incubation times, the nerve cords were fixed with 4% paraformaldehyde at RT for 1 h. After fixation, tissues were washed 3 times in PBS, permeabilized by a 24 h incubation at 4 °C in permeabilization solution (1% Triton X100 in PBS) and pre-incubated in blocking buffer (in 1% Triton, 3% Normal Donkey Serum (NDS) and 1% ovalbumin in PBS/glycine 0.1 M) for 8 h at 4 °C. Then the samples were incubated overnight at 4 °C with the appropriate primary antibody diluted in blocking buffer. After 3 washes with PBS, samples were incubated 1 h at 37 °C with secondary donkey anti-rabbit antibody conjugated to Alexa Fluor 488 (1:2000, Invitrogen, Carlsbad CA, USA) in blocking buffer. They were rinsed with PBS and the cell nuclei were counterstained by Hoechst 33342 fluorescent dye (1/10000, Invitrogen, Carlsbad CA, USA) for 20 min at 4 °C. Finally the nerve cords were mounted on the slide with Dako Fluorescent Mounting Medium (Agilent, Santa Clara CA, USA). Samples without the addition of primary antibody were used as negative control. Slides were maintained, observed and processed in the same manner as described above. The presented pictures are representative of independent triplicates.

### Chemotaxis assays

*In vitro* chemotaxis assays were performed by using the double-P assay as described by Köhidai, with minor modifications^65^ (Supplementary Method S5). Experiments were performed in triplicates. The results were expressed as the mean percentage of cells migrated, taking into account the starting amount as 100 percent ± S.E.D. Comparisons between means were made using the Ordinary one-way Anova using GraphPad Prism 6.0 software. Statistical differences were considered significant if p was <0.05.

Either recombinant form of human TGF-β (H8541, Sigma-Aldrich, Saint Louis MO, USA) (0, 0.5, 1, 5 and 10 ng/ml) or conditioned media (CM) containing their respective secretome (microglial cells and neurons collected for 15 min primary cultures, see below) were used as chemotactic factors. CM-dependent chemotaxis assays were performed also with neutralizing antibodies. Microglial cells were preincubated for 1 hour at RT with rabbit polyclonal anti-ALK5 antibody (1/100, ab125310, Abcam Cambridge, UK) to inhibit the receptor prior to assays. In another condition, neuron-conditioned medium was preincubated for 1 hour at RT with rabbit polyclonal anti-human TGF-β1 antibody (1/100, ab92486, Abcam, Cambridge, UK) to neutralize nGDF prior to assays. In every experiment, negative controls were carried out with complete L-15 medium alone.

### *Ex vivo* microglial cell recruitment assays

The experiment was performed in injured leech connectives (as described above) at different times post-injury (T6 h, T16 h and T24 h) perfused with a rabbit polyclonal anti-ALK5 (1/50, ab125310, Abcam, Cambridge, UK) or with a control rabbit IgG (1/50, SC2027, Santa Cruz TX, USA) at the same dilution. The antibodies were respectively injected (8 μL) inside the 2^nd^ and 3^rd^ ganglia in a 4 ganglia fragment. The connectives were crushed immediately after injection with fine forceps in the middle side of the two ganglia injected and the tissues were fixed in 4% paraformaldehyde, pH 7.4 for 1 h after T6 h, T16 h and T24 h. Microglial cells recruitment was followed by using a nuclear fluorescent dye Hoechst 33342 (1:10000, Invitrogen, Carlsbad CA, USA) for 20 min at 4 °C. Microglial cell movement in response to these different injections was then observed and processed in the same manner as described above. The presented pictures are representative of independent triplicates.

### Western blotting

CNS, microglial cells and neurons protein extract analysis were performed from 5 and 10 nerve cords respectively T24 h post-injury with RIPA buffer. For each experimental condition, SDS-PAGE was conducted (4–12% polyacrylamide gel) using 30 µg of protein extract homogenized (v/v) in 2X Laemmli sample buffer and loaded in the gel wells, further details are found in Supplementary Method S6. After migration of proteins the gel is transferred in a membrane and was incubated for 1 hour at RT in blocking buffer (0.05% Tween 20 w/v, 5% milk powder w/v in 0.1 M PBS, pH 7.4) and then overnight at 4 °C in rabbit polyclonal anti-TGF-β1 (1/200, Abcam, Cambridge, UK) in blocking buffer. After rinsing three times with PBS-0.05% Tween 20 for 15 minutes, the membrane was incubated for 1 hour at RT in secondary goat anti-rabbit polyclonal antibody conjugated with horseradish peroxidase (1:20,000, Jackson Immunoresearch, Cambridgeshire, UK) in PBS-0.05% Tween 20. Finally, after another rinsing with PBS, immunoreactive bands were revealed using the ECL Kit SuperSignal West Dura ChemoLuminescent Substrate (Thermo Fisher Scientific, Waltham MA, USA). Chemiluminescence analyses were performed by ImageQuant LAS-4000 mini system (Fujifilm, Tokyo, Japan).

### *In situ* micro-extraction of proteins

A large-scale proteomic analysis was developed on the injured connectives from the leech nerve cord. This spatially- and temporally-resolved proteomic study was performed on organotypic culture of isolated fragments of nerve chain respecting the integrity of several ganglia joined by connective tissues. Protein micro-extraction experiments were performed using the TriVersa Nanomate platform (Advion BioSciences, Ithaca NY, USA), with the Liquid Extraction Surface Analysis (LESA) feature^66^ with several modifications as previously described^67^. For our study, a CNS fragment composed by 4 leech ganglia is dissected. Then the leech nerve cord is injured by cutting one of the two connectives between each pair of ganglia (3 extraction points per fragment). The fragments were incubated in complete medium with a specific ALK4/5 inhibitor, SB431542 (20 µM, R&D Systems, Minneapolis MN, USA) or with its vehicle (100 mM DMSO). The fragments were mounted on the Poly-D-lysine slide (Dominique Dutscher, Brumath, France) at different times post-injury 15 minutes (T0), 6 hours (T6 h) and 24 hours (T24 h). The complete details of the LESA method are found in Supplementary Method S7. Proteins coming from cells and intercellular spaces are then extracted and stored in a collection tube. For proteomics analysis, the sample was migrated on an acrylamide gel. Then, the gels band were cut and subjected in gel digestion were the proteins underwent reduction, alkylation and enzymatic digestion (See Supplementary Method S8).

### NanoLC-HR-MS/MS

The samples were separated by online reversed-phase chromatography using a Proxeon EasynLC1000 system (Thermo Fisher Scientific, Waltham MA, USA) equipped with a Proxeon trap column (100 µm ID 2 cm) and a C18 packed-tip column (Acclaim PepMap, 75 µm ID 50 cm). Peptides were separated using an increasing amount of acetonitrile (5–30% over 120 min) at a flow rate of 300 nL/min. The chromatography system was coupled to a Q-exactive mass spectrometer (Thermo Fisher Scientific, Waltham MA, USA) programmed to acquire the top 10 MSMS in data-dependent mode. All the details of the NanoLC-HR-MS/MS can be found in Supplementary Method S9.

### Data analyses

All the MS data were processed with MaxQuant (version 1.5.6.5) software^68^ using the Andromeda search engine^69^. Proteins were identified by searching MS and MS/MS data against a homemade database of *Hirudo medicinalis* All the predicted protein sequences are annotated by homology with the human database. For identification, the FDR at the peptide spectrum matches (PSMs) and protein level was set to 0.01. Label-free quantification of proteins was performed using the MaxLFQ algorithm integrated into MaxQuant with the default parameters. Analysis of the proteins identified were performed using Perseus (version 1.5.6.0) software^70,71^. Multiple-samples tests were performed using ANOVA test with a p-value of 5% and preserving grouping in randomization. Visual heatmap representations of significant proteins were obtained using hierarchical clustering analysis. Functional annotation and characterization of identified proteins were obtained using PANTHER (version 13.0) software^72^ and STRING (version 9.1)^73^. The analysis of gene ontology, cellular components and biological processes, were performed with FunRich 3.0 analysis tool^74^. The details regarding the data analysis are found in Supplementary Method S10.

## Supplementary information

Supplementary informations

## Data Availability

The datasets generated during and/or analysed during the current study are available from the corresponding author on reasonable request.
