# Supplementary material for: ALK4/5-dependent TGF-β signaling contributes to the crosstalk between neurons and microglia following axonal lesion
Source: Sci Rep. 2019 May 3;9:6896. doi: 10.1038/s41598-019-43328-x (PMC6499822; doi:10.1038/s41598-019-43328-x)
Supplement: Supplementary file 1 — Supplementary informations [file 41598_2019_43328_MOESM1_ESM.docx]

**Title: ALK4/5-dependent TGF-β signaling contributes to the crosstalk between neurons and microglia following axonal lesion**

**Antonella RAFFO-ROMERO^1,2^, Tanina ARAB^1,2^, Christelle VAN CAMP, Quentin LEMAIRE^1,2^, Maxence WISZTORSKI^1,2^, Julien FRANCK^1,2^, Soulaimane ABOULOUARD^1,2^, Francoise LE MARREC-CROQ^1,2^, Pierre-Eric SAUTIERE^1,2^, Jacopo VIZIOLI^1,2^, Michel SALZET^1,2^ & Christophe LEFEBVRE^1,2*^**

^1^Univ. Lille, Inserm, U-1192 - Laboratoire Protéomique, Réponse Inflammatoire et Spectrométrie de Masse-PRISM, F-59000 Lille, France

^2^EURON – European Graduate School of Neuroscience, Maastricht, The Netherlands

*Corresponding author: Pr. Christophe Lefebvre, Univ. Lille, INSERM, U1192 - Laboratoire Protéomique, Réponse Inflammatoire et Spectrométrie de Masse-PRISM, F-59000 Lille, France, <http://www.laboratoire-prism.fr/>
Email: [christophe.lefebvre@univ-lille.fr](mailto:christophe.lefebvre@univ-lille.fr)
Tel : +33-320-434-127

Supplementary Information includes ten Supplementary Methods (Supplementary Methods S1-S10), three Supplementary Figures (Supplementary Figure S1 related to Figure 6; Supplementary Figure S2 related to Figure 7; Supplementary Figure S3 related to Figures 4 and 7) and 5 Supplementary Tables (Supplementary Table S1-5).

**Supplementary Method S1.**

**Leech CNS-Conditioned Medium (CM) for chemotaxis experiments**

For the preparation of conditioned medium from microglial cells and neurons, 10 nerve cords were dissected and cells were separated as previously described. Then, they were placed in fresh complete medium (500 µL) for 15 minutes (T0) and centrifuged for 20 min at 1,200 × g to eliminate microglial cells or neurons. The cell-free supernatant was then used as CM in the chemotaxis experiments.

**Supplementary Method S2.**

**RNA extraction**

Regarding the molecular characterization part of the material and methods, the total RNA have been extracted from the complete nerve cords corresponding to 10 leeches, then incubated in TRIzol® reagent (Thermo Fisher Scientific, Waltham MA, USA) and homogenized using Precellys® tissue homogenizer (Ozyme, Montigny-le-Bretonneux, France). For the gene expression analyses part, the total RNA have been extracted from neurons from the nerve cords of 10 leeches, then incubated in TRIzol® reagent (Thermo Fisher Scientific, Waltham MA, USA). The extraction of the total RNA was performed according to manufacturer’s protocol (Thermo Fisher Scientific, Waltham MA, USA) and resuspended in RNase-free water. The extracted total RNAs were treated with RQ1-DNase1 (Promega, Madison WI, USA) to prevent any contamination by genomic DNA.

**Supplementary Method S3.**

**RACE-PCR**

RACE-PCR was conducted using SMARTer® RACE 5’/3’ Kit (Takara, Kusatsu, Japan). Briefly, 2 µg of total RNA were used to construct 5’ and 3’ cDNA libraries according to the manufacturer’s instructions. Then Nested PCR ampliﬁcation reactions respecting Takara instructions were performed using a combination of forward and reverse primers deduced from TGF-β type I receptor putative partial mRNA sequence (Fw1 5’-AATAATCTTCGTCCTCTTGCTTCT-3’; Fw2 5’- TGGTGCCAGCATAGAAAGGTCC-3’; Rv1 5’-CACTGCCCACTCTGTTGTTG-3’; Rv2 5’-CGACTTTATCTGACTCTGAGTCA-3’) and from that of TGF-β1 (Fw1 5’-GTGGTTCTCGGACTCTCAAACGC-3’; Fw2 5’-CGGCATCAGAATGCCCAACCTG-3’; Rv1 5’-TCGCCCGTACACGATCTTCATCC-3’; Rv2 5’-GGCACAGCACATGCCTTTGTATTT-3’). The reaction cycles were performed as follows: 94°C for 1 min, followed by 40 cycles of 30 s at 94°C, 30 s at 56°C, and 2 min at 72°C.

**Supplementary Method S4.**

**Hybridization protocol**

Fixed CNS were subjected to four washes of 5 min each in PBT and then permeabilized for 10 min by digestion with 10 µg/ml Proteinase K (Sigma-Aldrich, Saint Louis MO, USA) in PBS. Then, they were washed 5 min in PBT two times, post-fixed for 25 min in 4% paraformaldehyde at RT and washed four times for 5 min each in PBT. They were incubated for 10 min in mixture 50% PBT/50% Hybe^(-)^ (50% (v/v) formamide, 5X SSC, 0.1 % (h/v) Tween 20 and sterile water pH 6.5) and then for 3 hours in Hybe^(-)^ at 42 °C.

Hybridization was performed overnight at 42 °C with riboprobes in Hybe^(+)^ (50 % (v/v) formamide, 5X SSC, 0.1 % (h/v) Tween 20, 500 µg/ml tRNA, 50 µg/ml heparin and H_2_O). After hybridization, nerve cords were washed through the following series at 42°C : two times for 5 min and three times for 20 min in Hybe^(-)^, then at RT 10 min in a mixture 50% PBT/50% Hybe^(-)^ and four times for 5 min in PBS/0.2 M glycine.

Nerve cords followed an incubation with blocking reagent 1/5 in PBT (Roche Diagnostics, Risch-Rotkreuz, Swiss) for 30 min and then incubated with a sheep polyclonal anti-DIG antibody 1/700 (abcam64509, Abcam, Cambridge, UK) for 2 hours at RT. After 4 washings in PBT for 10 min and incubation for 30 min in a solution blocking reagent 1/5 in PBT, samples were incubated with the secondary donkey anti-sheep antibody conjugated to Alexa Fluor 488 (1:1500, Invitrogen, Carlsbad CA, USA) in blocking reagent (Roche Diagnostics, Risch-Rotkreuz, Swiss) and rinsed with PBT before mounting with Fluorescent Mounting Medium (Agilent, Santa Clara CA, USA). The cell nuclei were counterstained by Hoechst 33342 fluorescent dye (1:10000, Invitrogen, Carlsbad CA, USA) for 20 min. The nerve cords were mounted on the slide with Dako Fluorescent Mounting Medium (Agilent, Santa Clara CA, USA).

**Supplementary Method S5.**

**Chemotaxis assays**

The chemotaxis assays experiments were performed using Petri dishes with 35 millimeter diameter. The latter were filled with 4 ml of 1% agar and 0.5% gelatin solution. After drying, two 6 mm diameter wells were made, each one presenting a parallel individual channel. One well was filled with 100 μl of purified microglial cells (see above) and the second was filled with chemotactic factors or negative controls. A channel was further created perpendicularly to others using a coverslip. One hour later, cells in the well containing chemoattractant were collected. The number of migrating cells was counted on a hemocytometer (five different counts) under Axioskop microscope (Zeiss, Oberkochen, Germany).

**Supplementary Method S6.**

**Western blotting**

CNS, microglial cells and neurons protein extract analysis were performed from 5 and 10 nerve cords respectively T24 h post-injury with RIPA buffer (150 mM NaCl, 50 mM Tris, 5 mM EGTA, 2 mM EDTA, 100 mM NaF, 10 mm sodium pyrophosphate, 1% Nonidet P-40, 1 mM PMSF, 1X protease inhibitors). Cell debris was removed by centrifugation (1,200 × g for 20 min, RT); the supernatants were collected and the protein concentrations were measured using a Protein Assay (BioRad, Hercules CA, USA).For each experimental condition, SDS-PAGE was conducted with 4-12% polyacrylamide gel. Protein extract (30 µg) was homogenized (v/v) in 2X Laemmli sample buffer and loaded on the gel. Migration was performed in TGS buffer pH 8.5 (25 mM Tris, 192 mM glycine, and 0.1% SDS). The gel was run at 70 V for 15 min and at 120 V for 45 min. The separated proteins were transferred to AmershamTM HybondTM-ECL nitrocellulose membranes (GE Healthcare, Little Chalfont, UK).

**Supplementary Method S7.**

**LESA method**

The fragments were mounted on the Poly-D-lysine slide (Dominique Dutscher, Brumath, France) at different times post-injury 15 minutes (T0), 6 hours (T6 h) and 24 hours (T24 h) and observed by microscopy to mark the lesion points. The glass slide is then mounted on a universal adapter plate and the precise X–Y position of the extraction site were determined using LESA Points software. A volume of 1.5 μl of CHAPS solution (3.5% in 0.1M Tris HCl pH 10 and 50mM DTT) was loaded into a tip. A robotic arm moved the tip above the extraction point previously determine. A liquid microjunction is formed between the end of the tip and the lesion point with a volume of 0.4 μl of solvent. Ten cycles of aspiration/dispense are performed on the lesion without breaking the liquid junction. Proteins coming from cells and intercellular spaces are then extracted and stored in a collection tube. For proteomics analysis, the sample is deposited on an acrylamide gel and subsequently subjected to reduction, alkylation and enzymatic digestion.

**Supplementary Method S8.**

**In gel Digestion**

Band gels were cut into small pieces of 1mm3 and then washed with 300 μL of distilled deionized water for 15 min, 300 μL of ACN for 15 min, 300 μL of NH4HCO3 (100mM, pH8) for 15 min, 300 μL of NH4HCO3/ACN (1:1, v/v) for 15 min and then 300 μL of ACN for 5 min. Band gel pieces were dried under vacuum using a speedvac for 5 min. Reduction of cysteines was performed using 50 µL of a solution of DTT (10mM) in NH4HCO3 (100 mM, pH8) and incubated at 56°C for 1 h. Alkylation of cysteines was performed using 50 μL of a solution of IAA (50mM) in NH4HCO3 (100 mM, pH8) and incubated at room temperatureRT in the dark for 30 min. Band gel pieces were washed with 300 μL of NH4HCO3 (100 mM, pH8) for 15 min, 300 μL of NH4HCO3/ACN (1:1, v/v) for 15 min and 300 μL of ACN for 5 min. Band gel pieces were then dried in a speedvac for 5min and subjected to enzymatic digestion using a solution of trypsin (12,5 μg/mL) in NH4HCO3 (20 mM, pH8) and incubated overnight at 37°C. Peptides were subsequently extracted using 50 μL of acetonitrile (ACN), 150 µL of 1% formic acid and then 150 μL of ACN. Supernatants were transferred in a new tube and dried using a speedvac. Peptides were then resuspended in 20 μL of a solution of 0.1% TFA for a subsequent desalting step.

**Supplementary Method S9.**

**NanoLC-HR-MS/MS**

Peptides were desalted and concentrated using a C18 ZipTip (Millipore, Saint-Quentin-en-Yvelines, France), eluted by 80% ACN and dried under vacuum. Dried samples were reconstituted in 20µL of ACN/0.1% aqueous FA (2:98, v/v). The samples were separated by online reversed-phase chromatography using a Proxeon EasynLC1000 system (Thermo Fisher Scientific, Waltham MA, USA) equipped with a Proxeon trap column (100 µm ID 2 cm) and a C18 packed-tip column (Acclaim PepMap, 75 µm ID 50cm). Peptides were separated using an increasing amount of acetonitrile (5–30% over 120 min) at a flow rate of 300 nL/min. The LC eluent was electrosprayed directly from the analytical column and a voltage of 1.7 kV was applied via the liquid junction of the nanospray source. The chromatography system was coupled to a Q-exactive mass spectrometer (Thermo Fisher Scientific, Waltham MA, USA) programmed to acquire the top 10 MSMS in data-dependent mode. The survey scans were done at a resolving power of 70,000 FWHM (m/z 400), in positive mode and using an AGC target of 3 x 10^6^. Default charge state was set at 2, unassigned and 1 charge states were rejected and dynamic exclusion was enabled for 25 s. The scan range was set to 300–1600 m/z. For ddMS2, the scan range was between 200–2000 m/z, 1 microscan was acquired at 17,500 FWHM, an isolation window of 4.0 m/z and a HCD Normalized Collision Energy (NCE) of 30 was used.

**Supplementary Method S10**

**Data analyses**

All the MS data were processed with MaxQuant (version 1.5.6.5) software using the Andromeda search engine. Proteins were identified by searching MS and MS/MS data against a homemade database of Hirudo medicinalis. This homemade database is based in translated mRNA sequences created from a H. medicinalis draft genome, as it was already described before. All the predicted protein sequences are annotated by homology with the human database. Trypsin specificity was used for the digestion mode with N-terminal acetylation and methionine oxidation selected as the variable. Carbarmidomethylation of cysteines was set as a fixed modification, with up to two missed cleavages. For MS spectra, an initial mass accuracy of 6 ppm was selected, with a minimum of 2 peptides and at least 1 unique peptide per protein, and the MS/MS tolerance was set to 20 ppm for HCD data. For identification, the FDR at the peptide spectrum matches (PSMs) and protein level was set to 0.01. Label-free quantification of proteins was performed using the MaxLFQ algorithm integrated into MaxQuant with the default parameters. Analysis of the proteins identified were performed using Perseus (version 1.5.6.0) software. The file containing the information from identification was used with hits to the reverse database, and proteins only identified with modified peptides and potential contaminants were removed. Then, the LFQ intensity was logarithmized (log2[x]). Categorical annotation of rows was used to define different groups after grouping replicates.

Multiple-samples tests were performed using ANOVA test with a p-value of 5% and preserving grouping in randomization. Visual heatmap representations of significant proteins were obtained using hierarchical clustering analysis. Normalization was achieved using a Z-score with a matrix access by rows. For the statistical analysis, only proteins presenting as significant by the ANOVA test were used. Hierarchical clustering depending protein extract were first performed using the Euclidean parameter for distance calculation and average option for linkage in row. Functional annotation and characterization of identified proteins were obtained using PANTHER (version 13.0) software and STRING (version 9.1). Integrated Venn diagram analysis was performed using “Draw venn diagram”; a web-based tool for the analysis of complex data sets. The analysis of gene ontology, cellular components and biological processes, were performed with FunRich 3.0 analysis tool.

**Supplementary Figure S1. ALK4/5-dependent protein signatures in the point of lesion.** (**a**) Venn Diagrams of the numerical values for common and exclusive proteins present in time-course (T0 in blue, T6h in pink and T24h in green) are represented separately for SB431542 inhibitor and for vehicle conditions. (**b**) MaxQuant and Perseus softwares were used for the statistical analysis of common protein signatures to generate heatmaps showing overexpressed proteins that were significantly different in T0 vs. T6h vs. T24h in SB431542 inhibitor as well as vehicle conditions. Three clusters in the control condition (vehicle) and four clusters in the inhibitor condition are highlighted (yellow). (**c**) Analysis of Biological pathway Gene Ontology (GO) terms focused on TGF-β signaling. The proteins detected in the cluster 1 (T0) from SB431542-dependent condition have been collected and compared to those of the cluster 1 (T0) from vehicle-dependent condition using TGF-β signaling-related GO terms in FunRich software. The graph shows the percentage of proteins identified by mass spectrometry that match into this GO term pathway relative to the total number of proteins identified. (**d**) Network shows proteins into GO extracellular exosome category (red) at T6h post-lesion (vehicle condition) using STRING database.

**Supplementary Figure S2. Microglial production of nGDF and its influence in neurons.** (**a-c**) Confocal microscopy analysis of nGDF immunofluorescence in lesioned nerve cord 24h post-injury using rabbit polyclonal anti-TGF-β antibodies with or without any ligature between connectives and ganglia (see diagrams). Some microglia and intercellular spaces are stained in ganglia (**a**, arrows) whereas conditions with ligatures preventing any relation between connectives and ganglia show no signal (**c**). (**b**) No signal was detected in tissues treated only with secondary antibody as negative control. Cell nuclei were stained with Hoechst 33342 (blue).

**Supplementary Figure S3. Preparation of Figure 4c (top) and Figure 7h (bottom) from western blot original image.** In the original image, membranes from the blot with primary and secondary antibodies (left) or blot with secondary antibody alone (right) were bridged at the molecular weight marker for imaging. The boxes describe the areas selected for the preparation of each image.

**Supplementary Table S1:** List of exclusive proteins represented in the Venn diagrams (shown in Supplementary Figure S1a) corresponding to Perseus analysis generated from the samples of different post-injury times with SB431542 or vehicle incubation separately.

| **Vehicle Exclusives** | | | **Inhibitor Exclusives** | | |
| --- | --- | --- | --- | --- | --- |
| **T0** | **T6h** | **T24h** | **T0** | **T6h** | **T24h** |
| **HGS (O14964)** | **COL6A3 (P12111)** | **ACLY (P53396)** | CRYZ (Q08257) | LGR5 (O75473) | ANK3 (Q12955) |
| GMPPB (Q9Y5P6) | **ANXA7 (P20073)** | CRYZ (Q08257) | ATP1B1 (P05026) | **PSMB8 (P28062)** | **HRSP12 (P52758)** |
| ATIC (P31939) | **ANPEP (P15144)** | **ISOC2 (Q96AB3)** | **CST4 (P01036)** | **AHNAK (Q09666)** | LAMA2 (P24043) |
| BLOC1S6 (Q9UL45) | **MAP1S (Q66K74)** | **CTSL (P07711)** | GMPPB (Q9Y5P6) | UGP2 (Q16851) | PMPCB (O75439) |
| **HMOX2 (P30519)** | **NDUFV2 (P19404)** | LAMA2 (P24043) | UGP2 (Q16851) | **KLHDC8A (Q8IYD2)** | DYNC1H1 (Q14204) |
| LCT (P09848) | DLAT (P10515) | LGR5 (O75473) | **SAR1B (Q9Y6B6)** | GNAQ (P50148) | **CPNE2 (Q96FN4)** |
| **SLC44A4 (Q53GD3)** | **PNP (P00491)** | PSMA8 (Q8TAA3) | **SBF2 (Q86WG5)** | DLAT (P10515) | **EHD3 (Q9NZN3)** |
| **SBSPON (Q8IVN8)** | **PHYH (O14832)** | **ALDH4A1 (P30038)** | ATIC (P31939) | **MYH3 (P11055)** | ALDH18A1 (P54886) |
| MDH2 (P40926) | **GNB2L1 (P63244)** | DYNC1H1 (Q14204) | PSMA8 (Q8TAA3) | **AARS (P49588)** | **PRMT1 (Q99873)** |
| ANK2 (Q01484) | **PCYT2 (Q99447)** | ANK3 (Q12955) | **SYAP1 (Q96A49)** | **YKT6 (O15498)** | ACO2 (Q99798) |
| GMPR2 (Q9P2T1) | LMNA (P02545) | ASAH1 (Q13510) | BLOC1S6 (Q9UL45) | **PTGES3 (Q15185)** | **HIST4H4 (P62805)** |
| NAMPT (P43490) | **RAP2C (Q9Y3L5)** | PLEC (Q15149) | **GLA (P06280)** | **SEPHS1 (P49903)** | **NT5C2 (P49902)** |
| MGAT5B (Q3V5L5) | **MAP3K20 (Q9NYL2)** | **OAT (P04181)** | **PTS (Q03393)** | **PDXK (O00764)** | ADPRH (P54922) |
| RAB18 (Q9NP72) | **AQP4 (P55087)** | GNAQ (P50148) | **PSMA6 (P60900)** | **AIMP1 (Q12904)** | **CALR (P27797)** |
| CNN1 (P51911) | **PYCR1 (P32322)** | DPYSL4 (O14531) | LCT (P09848) | **RPS14 (P62263)** |  |
| **NID1 (P14543)** | **SELENBP1 (Q13228)** | **TOM1L2 (Q6ZVM7)** | ASAH1 (Q13510) | **PSMB5 (P28074)** |  |
| PFKL (P17858) | **COL6A5 (A8TX70)** | **ACAT2 (Q9BWD1)** | **GRK1 (Q15835)** | **FAH (P16930)** |  |
| GLUL (P15104) | **RAP1B (P61224)** | **FKBP1A (P62942)** | **HARS (P12081)** | **PSMD7 (P51665)** |  |
| PSMA4 (P25789) | **DPYS (Q14117)** | ACO2 (Q99798) | MDH2 (P40926) | **CLIC4 (Q9Y696)** |  |
| ANK3 (Q12955) | **HIRA (P54198)** | **MINPP1 (Q9UNW1)** | **RAB1A (P62820)** | **P4HB (P07237)** |  |
| **PRKCSH (P14314)** | **PAICS (P22234)** | **NT5C2 (P49902)** | ANK2 (Q01484) | **ACTN1 (P12814)** |  |
| TCEB1 (Q15369) | **ATP2B2 (Q01814)** | DHX9 (Q08211) | PLEC (Q15149) |  |  |
| GOT1 (P17174) | **GRIA1 (P42261)** | **CDON (Q4KMG0)** | **GCAT (O75600)** |  |  |
| **ARPC5L (Q9BPX5)** | **GCLC (P48506)** | PMPCB (O75439) | **HSPA8 (P11142)** |  |  |
| PIP (P12273) | **BPNT1 (O95861)** | **NDUFS7 (O75251)** | GMPR2 (Q9P2T1) |  |  |
| PSMD13 (Q9UNM6) | **UBA52 (P62987)** | **PSMD2 (Q13200)** | NAMPT (P43490) |  |  |
| **RAB35 (Q15286)** | **ATP1A1 (P05023)** | **CCT3 (P49368)** | MGAT5B (Q3V5L5) |  |  |
| **ATG3 (Q9NT62)** | ATP1A3 (P13637) |  | **ACHE (P22303)** |  |  |
| DNAJB1 (P25685) | **GCH1 (P30793)** |  | **MVP (Q14764)** |  |  |
| NCS1 (P62166) | **ACTN3 (Q08043)** |  | **37500 (Q15019)** |  |  |
| **GLUD2 (P49448)** | **ACTC1 (P68032)** |  | RAB18 (Q9NP72) |  |  |
| **CAT (P04040)** | **CPNE6 (O95741)** |  | **CNPY4 (Q8N129)** |  |  |
| **CHL1 (O00533)** | ATP1B1 (P05026) |  | CNN1 (P51911) |  |  |
| **SLC9A3R2 (Q15599)** | **AKR1A1 (P14550)** |  | PFKL (P17858) |  |  |
| **RRAS2 (P62070)** | **TPRG1L (Q5T0D9)** |  | **ATP5J2 (P56134)** |  |  |
| LHPP (Q9H008) | **IMPA1 (P29218)** |  | PSMA4 (P25789) |  |  |
| ADCY9 (O60503) | **ANXA5 (P08758)** |  | **KIAA1161 (Q6NSJ0)** |  |  |
| ADPRH (P54922) | **CNTN1 (Q12860)** |  | **DCTN1 (Q14203)** |  |  |
| PAK3 (O75914) | **QARS (P47897)** |  | DPYSL4 (O14531) |  |  |
|  | **FLNB (O75369)** |  | TCEB1 (Q15369) |  |  |
|  | **APRT (P07741)** |  | GOT1 (P17174) |  |  |
|  | **RAB8A (P61006)** |  | **HYOU1 (Q9Y4L1)** |  |  |
|  | **UBE2L3 (P68036)** |  | **ARRB1 (P49407)** |  |  |
|  | **ATP5H (O75947)** |  | ATP1A3 (P13637) |  |  |
|  | **CHP1 (Q99653)** |  | **GMDS (O60547)** |  |  |
|  | **GBE1 (Q04446)** |  | **RBKS (Q9H477)** |  |  |
|  | **ATP6V1A (P38606)** |  | PIP (P12273) |  |  |
|  | **COX4I1 (P13073)** |  | PSMD13 (Q9UNM6) |  |  |
|  | **FLOT1 (O75955)** |  | **TPD52 (P55327)** |  |  |
|  | **ACAT1 (P24752)** |  | **PMM2 (O15305)** |  |  |
|  | **GDI1 (P31150)** |  | **ATP6V1G1 (O75348)** |  |  |
|  | **QSOX1 (O00391)** |  | **ATP2B1 (P20020)** |  |  |
|  | **TUBA1A (Q71U36)** |  | DNAJB1 (P25685) |  |  |
|  | **NPEPPS (P55786)** |  | **CAPZA1 (P52907)** |  |  |
|  | **GNGT1 (P63211)** |  | **PTPRN (Q16849)** |  |  |
|  | **DCTN3 (O75935)** |  | NCS1 (P62166) |  |  |
|  | **PSMA2 (P25787)** |  | **SKI (P12755)** |  |  |
|  | **TNIP2 (Q8NFZ5)** |  | **TBCA (O75347)** |  |  |
|  | ANK2 (Q01484) |  | **ECE1 (P42892)** |  |  |
|  | **ACO1 (P21399)** |  | LHPP (Q9H008) |  |  |
|  | ALDH18A1 (P54886) |  | ADCY9 (O60503) |  |  |
|  | **ADRBK1 (P25098)** |  | LMNA (P02545) |  |  |
|  | DYNC1H1 (Q14204) |  | DHX9 (Q08211) |  |  |
|  | **SRI (P30626)** |  | **NAP1L4 (Q99733)** |  |  |
|  | **CAPN3 (P20807)** |  | **PSMF1 (Q92530)** |  |  |
|  |  |  | **CCT8 (P50990)** |  |  |
|  |  |  | GLUL (P15104) |  |  |
|  |  |  | PAK3 (O75914) |  |  |
|  |  |  | **NDUFA10 (O95299)** |  |  |

**Supplementary Table S2:** List of overexpressed proteins identified in specific clusters after Perseus analyses (extracted from the two heatmaps shown in Supplementary Figure S1b) generated from the samples of different post-injury times with SB431542 or vehicle incubation separately.

| **Vehicle Heatmap** | | | **Inhibitor Heatmap** | | |
| --- | --- | --- | --- | --- | --- |
| **Cluster 1** | **Cluster 2** | **Cluster 3** | **Cluster 1** | **Cluster 2** | **Cluster 3** |
| TPM2 (P07951) | **HSPA5 (P11021)** | **NCAM2 (O15394)** | TPM2 (P07951) | **PEF1 (Q9UBV8)** | **LETM1 (O95202)** |
| HSP90AB1 (P08238) | EHD1 (Q9H4M9) | **TSPAN1 (O60635)** | **PRKAR2A (P13861)** | **CLYBL (Q8N0X4)** | **FLOT2 (Q14254)** |
| YWHAB (P31946) | HSPD1 (P10809) | EML1 (O00423) | **TSPAN7 (P41732)** | **SLC13A5 (Q86YT5)** | **PAPSS1 (O43252)** |
| **PABPC1 (P11940)** | GSN (P06396) | **HAGH (Q16775)** | NSF (P46459) | **RAB39A (Q14964)** | EML1 (O00423) |
| WDR1 (O75083) | **TPT1 (P13693)** | **PPP2CA (P67775)** | **PYGB (P11216)** | **HSPE1 (P61604)** | NEFL (P07196) |
| **FABP5 (Q01469)** | **LAP3 (P28838)** | **GNAO1 (P09471)** | EHD1 (Q9H4M9) | CDC42 (P60953) | **ACTA2 (P62736)** |
| RDX (P35241) | **DPYSL2 (Q16555)** | **PGK2 (P07205)** | **ATP5A1 (P25705)** | **ALDH18A1 (P54886)** | **ATP5B (P06576)** |
| VWA5A (O00534) | **ANK3 (Q12955)** | **RPS23 (P62266)** | NEFL (P07196) | **CMPK1 (P30085)** | **TNS1 (Q9HBL0)** |
| PYGM (P11217) | **CAP1 (Q01518)** | CDC42 (P60953) | HSP90AB1 (P08238) | **ACHE (P22303)** | **ACSS2 (Q9NR19)** |
| CSTB (P04080) | **GARS (P41250)** | **CRYZ (Q08257)** | **ANXA7 (P20073)** | **GSTA1 (P08263)** | **SLC25A31 (Q9H0C2)** |
| **TST (Q16762)** | **TPM1 (P09493)** | **NDUFS3 (O75489)** | YWHAB (P31946) |  | **SSUH2 (Q9Y2M2)** |
| **HSPA8 (P11142)** | **GSTA4 (O15217)** | NSF (P46459) | HSPD1 (P10809) |  |  |
| **TTC38 (Q5R3I4)** |  |  | **MDH2 (P40926)** |  |  |
| HSPA4L (O95757) |  |  | **AHCY (P23526)** |  |  |
| MAP1S (Q66K74) |  |  | **CAPN2 (P17655)** |  |  |
| GSN (P06396) |  |  | **RAB3A (P20336)** |  |  |
| **ST13 (P50502)** |  |  | **PDHB (P11177)** |  |  |
| GPI (P06744) |  |  | **OGDH (Q02218)** |  |  |
| **GNB4 (Q9HAV0)** |  |  | **EML2 (O95834)** |  |  |
| **AHSA1 (O95433)** |  |  | **CBR1 (P16152)** |  |  |
| PFN1 (P07737) |  |  | **PPP3CB (P16298)** |  |  |
| NCS1 (P62166) |  |  | **BFSP2 (Q13515)** |  |  |
| DBNL (Q9UJU6) |  |  | **MSN (P26038)** |  |  |
| **SARS (P49591)** |  |  | RDX (P35241) |  |  |
| SUCLA2 (Q9P2R7) |  |  | **DLD (P09622)** |  |  |
| REEP5 (Q00765) |  |  | VWA5A (O00534) |  |  |
| CAPNS1 (P04632) |  |  | PYGM (P11217) |  |  |
| **ARL8A (Q96BM9)** |  |  | **NFS1 (Q9Y697)** |  |  |
|  |  |  | CSTB (P04080) |  |  |
|  |  |  | **ATP1A3 (P13637)** |  |  |
|  |  |  | SUCLA2 (Q9P2R7) |  |  |
|  |  |  | **OGDHL (Q9ULD0)** |  |  |
|  |  |  | **ATP6V1E1 (P36543)** |  |  |
|  |  |  | **PDHA1 (P08559)** |  |  |
|  |  |  | **HSPA9 (P38646)** |  |  |
|  |  |  | **LLGL2 (Q6P1M3)** |  |  |
|  |  |  | **SUCLG1 (P53597)** |  |  |
|  |  |  | **SPTB (P11277)** |  |  |
|  |  |  | **SOD1 (P00441)** |  |  |
|  |  |  | **HSP90AA1 (P07900)** |  |  |
|  |  |  | **AMT (P48728)** |  |  |
|  |  |  | **GNAQ (P50148)** |  |  |
|  |  |  | HSPA4L (O95757) |  |  |
|  |  |  | **FH (P07954)** |  |  |
|  |  |  | **NDUFV1 (P49821)** |  |  |
|  |  |  | **VCP (P55072)** |  |  |
|  |  |  | **SDHD (O14521)** |  |  |
|  |  |  | MAP1S (Q66K74) |  |  |
|  |  |  | **CDC37 (Q16543)** |  |  |
|  |  |  | **BLOC1S6 (Q9UL45)** |  |  |
|  |  |  | **COL1A2 (P08123)** |  |  |
|  |  |  | **LASP1 (Q14847)** |  |  |
|  |  |  | **IDH2 (P48735)** |  |  |
|  |  |  | **PRDX2 (P32119)** |  |  |
|  |  |  | **PARK7 (Q99497)** |  |  |
|  |  |  | **ACAT1 (P24752)** |  |  |
|  |  |  | **PPIA (P62937)** |  |  |
|  |  |  | **SERPINB1 (P30740)** |  |  |
|  |  |  | **CAPSL (Q8WWF8)** |  |  |
|  |  |  | **UQCRFS1 (P47985)** |  |  |
|  |  |  | **SCCPDH (Q8NBX0)** |  |  |
|  |  |  | **ATP5C1 (P36542)** |  |  |
|  |  |  | **SDHA (P31040)** |  |  |
|  |  |  | GPI (P06744) |  |  |
|  |  |  | **CCT6A (P40227)** |  |  |
|  |  |  | **PPP2R1A (P30153)** |  |  |
|  |  |  | **TCP1 (P17987)** |  |  |
|  |  |  | PFN1 (P07737) |  |  |
|  |  |  | **IDH3A (P50213)** |  |  |
|  |  |  | **STXBP1 (P61764)** |  |  |
|  |  |  | **ADD1 (P35611)** |  |  |
|  |  |  | NCS1 (P62166) |  |  |
|  |  |  | **ANXA2 (P07355)** |  |  |
|  |  |  | **CALR (P27797)** |  |  |
|  |  |  | DBNL (Q9UJU6) |  |  |
|  |  |  | **YWHAE (P62258)** |  |  |
|  |  |  | **EIF5A (P63241)** |  |  |
|  |  |  | **CS (O75390)** |  |  |
|  |  |  | **ANXA13 (P27216)** |  |  |
|  |  |  | **ALDH2 (P05091)** |  |  |
|  |  |  | **EEF2 (P13639)** |  |  |
|  |  |  | **ATP1A2 (P50993)** |  |  |
|  |  |  | **BPNT1 (O95861)** |  |  |
|  |  |  | **MVP (Q14764)** |  |  |
|  |  |  | **ATP1A1 (P05023)** |  |  |
|  |  |  | **YWHAZ (P63104)** |  |  |
|  |  |  | **VDAC2 (P45880)** |  |  |
|  |  |  | REEP5 (Q00765) |  |  |
|  |  |  | CAPNS1 (P04632) |  |  |
|  |  |  | **MDH1 (P40925)** |  |  |

**Supplementary Table S3:** List of exclusive proteins represented in the Venn diagrams (shown in Figure 6b) corresponding to Perseus analysis generated from the samples of SB431542 vs. vehicle conditions time by time separately. The proteins involved in a biological pathway (Figure 6d) were tagged with different numbers in the table (❶ CXCR4-mediated signaling events, ❷ Neurotrophic factor-mediated Trk receptor signaling, ❸ Apoptosis, ❹ Cell-Cell communication, ❺ Axon guidance, ❻ Innate Immune System).

| **T0h** | | **T6h** | | **T24h** | |
| --- | --- | --- | --- | --- | --- |
| **Vehicle** | **Inhibitor** | **Vehicle** | **Inhibitor** | **Vehicle** | **Inhibitor** |
| **ABHD11 (Q8NFV4)** | **37500 (Q15019)** | **51776 (Q9NYL2)** | **AARS (P49588)** | ANK2 (Q01484) **❺** | ACHE (P22303) |
| **ADAD1 (Q96M93)** | ACHE (P22303) | **ACAT1 (P24752)** | **ACTN1 (P12814)** | DCTN1 (Q14203) | AHNAK (Q09666) |
| **ADPRH (P54922)** | **AHCY (P23526)** | **ACO1 (P21399)** | AHNAK (Q09666) | GCH1 (P30793) | FAH (P16930) |
| **ARPC5L (Q9BPX5)** | **AHCYL1 (O43865)** | **ACTC1 (P68032)** | **AIMP1 (Q12904)** | GLA (P06280) | KIAA1161 (Q6NSJ0) |
| **CAT (P04040)** | **AKR1A1 (P14550)** | **ACTN3 (Q08043) ❹** | ANK3 (Q12955) | GMDS (O60547) | **MGAT5B (Q3V5L5)** |
| **CBR1 (P16152)** | ANK2 (Q01484) **❺** | **ADRBK1 (P25098) ❶** | **ATG3 (Q9NT62)** | HARS (P12081) | NPEPPS (P55786) |
| **CD9 (P21926)** | ANK3 (Q12955) | **ALDH18A1 (P54886)** | ATP1B1 (P05026) | **LHPP (Q9H008)** |  |
| **CDC42 (P60953) ❶❷❺** | **ARPC3 (O15145)** | ANK2 (Q01484) **❺** | CAPZB (P47756) | MINPP1 (Q9UNW1) |  |
| **CHL1 (O00533) ❺** | **ASAH1 (Q13510)** | **ANPEP (P15144)** | **CDON (Q4KMG0)** | MVP (Q14764) |  |
| EHD3 (Q9NZN3) | ATP1A3 (P13637) | **ANXA5 (P08758)** | **CLIC4 (Q9Y696)** | **PSMD13 (Q9UNM6) ❸** |  |
| **ESYT2 (A0FGR8)** | **ATP5J2 (P56134)** | **ANXA7 (P20073)** | **CPVL (Q9H3G5)** | PTPRN (Q16849) |  |
| **FH (P07954)** | CAPZA1 (P52907) **❻** | **APRT (P07741)** | **CRYBG3 (Q68DQ2)** | PYCR1 (P32322) |  |
| **FLNA (P21333) ❶❹** | CAPZB (P47756) | **AQP4 (P55087)** | CTSL (P07711) |  |  |
| **HGS (O14964) ❶** | **CCT2 (P78371)** | **ARF1 (P84077)** | DLAT (P10515) |  |  |
| **HSD17B10 (Q99714)** | **CFL2 (Q9Y281)** | **ATP1A1 (P05023)** | **DYNLL2 (Q96FJ2) ❸** |  |  |
| **KIF5B (P33176)** | **CNPY4 (Q8N129)** | ATP1A3 (P13637) | **ENTPD5 (O75356)** |  |  |
| **MATN1 (P21941)** | CPNE7 (Q9UBL6) | ATP1B1 (P05026) | FAH (P16930) |  |  |
| **MIF (P14174)** | **CRYZ (Q08257)** | **ATP2B2 (Q01814)** | FKBP1A (P62942) **❶** |  |  |
| NSF (P46459) | **CST4 (P01036)** | **ATP5H (O75947)** | **FLNC (Q14315)** |  |  |
| PCK2 (Q16822) | CTSL (P07711) | **ATP6V1A (P38606)** | **GLUD2 (P49448)** |  |  |
| **PRKCSH (P14314) ❻** | DCTN1 (Q14203) | **ATP6V1G1 (O75348)** | GLUL (P15104) |  |  |
| **QDPR (P09417)** | **DHX9 (Q08211)** | **BPNT1 (O95861)** | **GNAQ (P50148)** |  |  |
| **SYNE1 (Q8NF91)** | **DPYS (Q14117)** | **CALR (P27797)** | **HMOX2 (P30519)** |  |  |
| **TUBA4A (P68366)** | **DPYSL4 (O14531) ❺** | **CAPN3 (P20807)** | HSPA12B (Q96MM6) |  |  |
|  | **ECE1 (P42892)** | **CAPN5 (O15484)** | **KLHDC8A (Q8IYD2)** |  |  |
|  | FKBP1A (P62942) **❶** | CAPZA1 (P52907) **❻** | **LASP1 (Q14847)** |  |  |
|  | GLA (P06280) | **CHP1 (Q99653) ❶** | **LGR5 (O75473)** |  |  |
|  | GLUL (P15104) | **CNTN1 (Q12860) ❺** | MAP1S (Q66K74) |  |  |
|  | GMDS (O60547) | **COL6A3 (P12111)** | MINPP1 (Q9UNW1) |  |  |
|  | **GPT2 (Q8TD30)** | **COL6A5 (A8TX70)** | **MYH3 (P11055)** |  |  |
|  | HSPA12B (Q96MM6) | **COX4I1 (P13073)** | NDUFS7 (O75251) |  |  |
|  | **HYOU1 (Q9Y4L1)** | **CPNE2 (Q96FN4)** | **NID1 (P14543)** |  |  |
|  | KIAA1161 (Q6NSJ0) | **CPNE6 (O95741)** | NSF (P46459) |  |  |
|  | **KLC2 (Q9H0B6)** | CPNE7 (Q9UBL6) | **OTOF (Q9HC10)** |  |  |
|  | **LAP3 (P28838)** | **DBNL (Q9UJU6) ❶❸** | **P4HB (P07237)** |  |  |
|  | LRP2 (P98164) | **DCTN3 (O75935)** | **PDXK (O00764)** |  |  |
|  | MINPP1 (Q9UNW1) | DLAT (P10515) | **PIN4 (Q9Y237)** |  |  |
|  | **MTHFD2L (Q9H903)** | **DYNC1H1 (Q14204)** | **PIP4K2A (P48426)** |  |  |
|  | MVP (Q14764) | **EHD1 (Q9H4M9)** | **PREP (P48147)** |  |  |
|  | **NDUFA10 (O95299)** | EHD3 (Q9NZN3) | **PRTFDC1 (Q9NRG1)** |  |  |
|  | **NDUFS2 (O75306)** | **FLNB (O75369)** | **PSMB1 (P20618) ❸** |  |  |
|  | NDUFS7 (O75251) | **FLOT1 (O75955)** | **PSMB5 (P28074) ❸** |  |  |
|  | **OAT (P04181)** | **GBE1 (Q04446)** | **PSMB8 (P28062) ❸** |  |  |
|  | **PAFAH1B2 (P68402)** | **GCAT (O75600)** | **PSMD7 (P51665) ❸** |  |  |
|  | PCK2 (Q16822) | GCH1 (P30793) | **PTGES3 (Q15185)** |  |  |
|  | **PLEC (Q15149) ❸** | **GCLC (P48506)** | **RAB35 (Q15286)** |  |  |
|  | **PMPCB (O75439)** | **GDI1 (P31150)** | RPLP0 (P05388) |  |  |
|  | **PPP2CA (P67775)** | **GNB2L1 (P63244) ❶** | **RPS14 (P62263)** |  |  |
|  | PSMA6 (P60900) **❸** | **GNGT1 (P63211)** | **RRAS2 (P62070)** |  |  |
|  | **PSMA8 (Q8TAA3) ❸** | **GRIA1 (P42261)** | **SBSPON (Q8IVN8)** |  |  |
|  | **PSMD2 (Q13200) ❸** | HARS (P12081) | **SDHD (O14521)** |  |  |
|  | **PSMF1 (Q92530) ❸** | **HECTD3 (Q5T447)** | **SEPHS1 (P49903)** |  |  |
|  | PTPRN (Q16849) | **HIRA (P54198)** | **SH3BGR (P55822)** |  |  |
|  | RPLP0 (P05388) | **IMPA1 (P29218)** | **SLC44A1 (Q8WWI5)** |  |  |
|  | **SAR1B (Q9Y6B6)** | LRP2 (P98164) | **SLC44A4 (Q53GD3)** |  |  |
|  | **SBF2 (Q86WG5)** | MAP1S (Q66K74) | **SLC9A3R2 (Q15599)** |  |  |
|  | **SKI (P12755)** | **MAPK14 (Q16539) ❶❷❹❻** | **TMSB15A (P0CG35)** |  |  |
|  | **SYAP1 (Q96A49)** | **NAP1L4 (Q99733)** | **TNS1 (Q9HBL0)** |  |  |
|  | **TBCA (O75347)** | **NDUFV2 (P19404)** | **TPPP2 (P59282)** |  |  |
|  | **TFG (Q92734)** | **NID2 (Q14112)** | UGP2 (Q16851) |  |  |
|  | **TMSB15A,B (P0CG35)** | NPEPPS (P55786) | **YKT6 (O15498)** |  |  |
|  | **TPD52 (P55327)** | **NUDC (Q9Y266)** |  |  |  |
|  | **VWA7 (Q9Y334)** | **PAICS (P22234)** |  |  |  |
|  |  | PCK2 (Q16822) |  |  |  |
|  |  | **PCYT2 (Q99447)** |  |  |  |
|  |  | **PDCD6IP (Q8WUM4)** |  |  |  |
|  |  | **PHYH (O14832)** |  |  |  |
|  |  | **PMM2 (O15305)** |  |  |  |
|  |  | **PNP (P00491)** |  |  |  |
|  |  | **PRKAR2A (P13861)** |  |  |  |
|  |  | **PSMA2 (P25787) ❸** |  |  |  |
|  |  | PSMA6 (P60900) **❸** |  |  |  |
|  |  | **PTS (Q03393)** |  |  |  |
|  |  | PYCR1 (P32322) |  |  |  |
|  |  | **QARS (P47897)** |  |  |  |
|  |  | **QSOX1 (O00391)** |  |  |  |
|  |  | **RAB1A (P62820)** |  |  |  |
|  |  | **RAB8A (P61006)** |  |  |  |
|  |  | **RAP1B (P61224) ❶❷❺** |  |  |  |
|  |  | **RAP2C (Q9Y3L5)** |  |  |  |
|  |  | **SELENBP1 (Q13228)** |  |  |  |
|  |  | **SRI (P30626)** |  |  |  |
|  |  | **STXBP1 (P61764)** |  |  |  |
|  |  | **TBCB (Q99426)** |  |  |  |
|  |  | **TCP1 (P17987)** |  |  |  |
|  |  | **THOP1 (P52888)** |  |  |  |
|  |  | **TNIP2 (Q8NFZ5)** |  |  |  |
|  |  | **TOM1 (O60784)** |  |  |  |
|  |  | **TPRG1L (Q5T0D9)** |  |  |  |
|  |  | **TUBA1A (Q71U36)** |  |  |  |
|  |  | **UBA52 (P62987) ❸❻** |  |  |  |
|  |  | **UBE2L3 (P68036)** |  |  |  |
|  |  | UGP2 (Q16851) |  |  |  |
|  |  | **YES1 (P07947) ❶** |  |  |  |

**Supplementary Table S4:** List of overexpressed proteins identified in specific clusters after Perseus analyses (extracted from the three heatmaps shown in Figure 6c) generated from the samples of SB431542 vs. vehicle conditions time by time separately. The proteins involved in a biological pathway (Figure 6d) were tagged with different numbers in the table (❶ CXCR4-mediated signaling events, ❷ Neurotrophic factor-mediated Trk receptor signaling, ❸ Apoptosis, ❹ Cell-Cell communication, ❺ Axon guidance, ❻ Innate Immune System).

| **T0h** | | **T6h** | | **T24h** | |
| --- | --- | --- | --- | --- | --- |
| **Vehicle** | **Inhibitor** | **Vehicle** | **Inhibitor** | **Vehicle** | **Inhibitor** |
| **AMPD3 (Q01432)** | **ADD1 (P35611) ❸** | **ACSS2 (Q9NR19)** | BPNT1 (O95861) | **ACADL (P28330)** | PPP3CB (P16298) |
| CRYBG3 (Q68DQ2) | **ATP5A1 (P25705)** | **ANK3 (Q12955)** | C21ORF33 (P30042) | **CMPK1 (P30085)** | **SSUH2 (Q9Y2M2)** |
| **ENOPH1 (Q9UHY7)** | BPNT1 (O95861) | **ANXA13 (P27216)** | **CYCS (P99999) ❸** | FLNC (Q14315) **❹** | **TPM2 (P07951)** |
| FLNC (Q14315) **❹** | C21ORF33 (P30042) | **BFSP2 (Q13515)** | **GSTA1 (P08263)** | **GYG1 (P46976)** |  |
| NARS (O43776) | CRYBG3 (Q68DQ2) | **CALCOCO2 (Q13137)** | **MAP1S (Q66K74)** | HSPA5 (P11021) |  |
| **RAB11A (P62491)** | **EML2 (O95834)** | **CALR (P27797)** | **MYH4 (Q9Y623)** | **HSPA8 (P11142)** |  |
| **TUBA4A (P68366)** | FLII (Q13045) | **CAP1 (Q01518) ❺** | **PEF1 (Q9UBV8)** | **NTRK2 (Q16620) ❷** |  |
| **VAT1L (Q9HCJ6)** | **GNAQ (P50148)** | **CS (O75390)** | **SCCPDH (Q8NBX0)** | **PCYT2 (Q99447)** |  |
| **XPNPEP1 (Q9NQW7)** | HSPD1 (P10809) | **EHD1 (Q9H4M9)** | SDHD (O14521) | **PDHA1 (P08559)** |  |
| **LCP1 (P13796)** | **MVP (Q14764)** | **ENTPD1 (P49961)** | **WDR1 (O75083)** | YWHAZ (P63104) **❷** |  |
| **CLYBL (Q8N0X4)** | **NDUFS1 (P28331)** | **FH (P07954)** | YWHAZ (P63104) ❷ |  |  |
|  | **NEFL (P07196)** | FLII (Q13045) |  |  |  |
|  | **OGDHL (Q9ULD0)** | **FSCN1 (Q16658)** |  |  |  |
|  | **PGK2 (P07205)** | **GNAO1 (P09471) ❶** |  |  |  |
|  | **PIP4K2A (P48426)** | **GPI (P06744)** |  |  |  |
|  | PPP3CB (P16298) | HSPA5 (P11021) |  |  |  |
|  | **PYGB (P11216)** | **HSPB1 (P04792)** |  |  |  |
|  | **RPS23 (P62266)** | HSPD1 (P10809) |  |  |  |
|  | **RSPH1 (Q8WYR4)** | **MDH2 (P40926)** |  |  |  |
|  | SDHD (O14521) | NARS (O43776) |  |  |  |
|  | **SPTBN1 (Q01082) ❺** | **NDUFS3 (O75489)** |  |  |  |
|  | SUCLA2 (Q9P2R7) | **NTRK2 (Q16620) ❷** |  |  |  |
|  | **SUCLG1 (P53597)** | **PFKL (P17858)** |  |  |  |
|  | **UQCRFS1 (P47985)** | **PKM (P14618)** |  |  |  |
|  | **YWHAQ (P27348) ❷** | **PKN1 (Q16512)** |  |  |  |
|  |  | **PRDX2 (P32119)** |  |  |  |
|  |  | **SDHA (P31040)** |  |  |  |
|  |  | SUCLA2 (Q9P2R7) |  |  |  |
|  |  | **TPM1 (P09493)** |  |  |  |
|  |  | **TPT1 (P13693)** |  |  |  |
|  |  | **TSPAN1 (O60635)** |  |  |  |
|  |  | **VWA5A (O00534)** |  |  |  |

**Supplementary Table S5:** List of exosome-related proteins identified into “Exosomes” GO category generated with exclusive (venn diagrams) and overexpressed proteins (heatmaps) from the samples of SB431542 vs. vehicle conditions time by time separately (shown in Figure 6e).

| **T0** | | **T6 h** | | **T24 h** | |
| --- | --- | --- | --- | --- | --- |
| **Vehicle** | **Inhibitor** | **Vehicle** | **Inhibitor** | **Vehicle** | **Inhibitor** |
| RAB11A (P62491) | PYGB (P11216) | EHD1 (Q9H4M9) | PEF1 (Q9UBV8) | CMPK1 (P30085) | AHNAK (Q09666) |
| NARS (O43776) | ATP5A1 (P25705) | FSCN1 (Q16658) | WDR1 (O75083) | HSPA8 (P11142) | FAH (P16930) |
| FLNC (Q14315) | SPTBN1 (Q01082) | HSPD1 (P10809) | GSTA1 (P08263) | HSPA5 (P11021) | NPEPPS (P55786) |
| TUBA4A (P68366) | HSPD1 (P10809) | TSPAN1 (O60635) | YWHAZ (P63104) | FLNC (Q14315) |  |
| LCP1 (P13796) | GNAQ (P50148) | HSPB1 (P04792) | SLC44A4 (Q53GD3) | YWHAZ (P63104) |  |
| FH (P07954) | YWHAQ (P27348) | HSPA5 (P11021) | PSMB8 (P28062) | MVP (Q14764) |  |
| FLNA (P21333) | SUCLA2 (Q9P2R7) | FH (P07954) | AHNAK (Q09666) | GMDS (O60547) |  |
| HGS (O14964) | MVP (Q14764) | ENTPD1 (P49961) | PSMB1 (P20618) | PSMD13 (Q9UNM6) |  |
| SYNE1 (Q8NF91) | AHCYL1 (O43865) | NARS (O43776) | CPVL (Q9H3G5) |  |  |
| CDC42 (P60953) | CRYZ (Q08257) | MDH2 (P40926) | UGP2 (Q16851) |  |  |
| EHD3 (Q9NZN3) | CST4 (P01036) | PFKL (P17858) | NID1 (P14543) |  |  |
| CD9 (P21926) | ARPC3 (O15145) | PRDX2 (P32119) | CAPZB (P47756) |  |  |
| CBR1 (P16152) | SYAP1 (Q96A49) | CAP1 (Q01518) | GNAQ (P50148) |  |  |
| ARPC5L (Q9BPX5) | AHCY (P23526) | GPI (P06744) | GLUL (P15104) |  |  |
| HSD17B10 (Q99714) | PSMA6 (P60900) | CALR (P27797) | FLNC (Q14315) |  |  |
| QDPR (P09417) | DPYS (Q14117) | PKM (P14618) | MYH3 (P11055) |  |  |
| MIF (P14174) | ASAH1 (Q13510) | SUCLA2 (Q9P2R7) | AARS (P49588) |  |  |
|  | CFL2 (Q9Y281) | CS (O75390) | PDXK (O00764) |  |  |
|  | PLEC (Q15149) | ANXA13 (P27216) | ATP1B1 (P05026) |  |  |
|  | CCT2 (P78371) | TPM1 (P09493) | RAB35 (Q15286) |  |  |
|  | SEPT2 (Q15019) | COL6A3 (P12111) | RPS14 (P62263) |  |  |
|  | CAPZB (P47756) | PRKAR2A (P13861) | PSMB5 (P28074) |  |  |
|  | AKR1A1 (P14550) | ANXA7 (P20073) | FAH (P16930) |  |  |
|  | LRP2 (P98164) | DBNL (Q9UJU6) | FKBP1A (P62942) |  |  |
|  | HYOU1 (Q9Y4L1) | ATP1B1 (P05026) | RPLP0 (P05388) |  |  |
|  | ATP1A3 (P13637) | ANPEP (P15144) | SLC44A1 (Q8WWI5) |  |  |
|  | GMDS (O60547) | TOM1 (O60784) | PSMD7 (P51665) |  |  |
|  | CAPZA1 (P52907) | UGP2 (Q16851) | SLC9A3R2 (Q15599) |  |  |
|  | FKBP1A (P62942) | PNP (P00491) | RRAS2 (P62070) |  |  |
|  | TBCA (O75347) | GNB2L1 (P63244) | CLIC4 (Q9Y696) |  |  |
|  | RPLP0 (P05388) | RAP2C (Q9Y3L5) | P4HB (P07237) |  |  |
|  | PPP2CA (P67775) | SELENBP1 (Q13228) | ACTN1 (P12814) |  |  |
|  | ECE1 (P42892) | PSMA6 (P60900) |  |  |  |
|  | PAFAH1B2 (P68402) | RAP1B (P61224) |  |  |  |
|  | DHX9 (Q08211) | HIRA (P54198) |  |  |  |
|  | LAP3 (P28838) | PAICS (P22234) |  |  |  |
|  | PSMD2 (Q13200) | ATP2B2 (Q01814) |  |  |  |
|  | GLUL (P15104) | TCP1 (P17987) |  |  |  |
|  |  | FLOT1 (O75955) |  |  |  |
|  |  | UBA52 (P62987) |  |  |  |
|  |  | RAB1A (P62820) |  |  |  |
|  |  | ATP1A1 (P05023) |  |  |  |
|  |  | ATP1A3 (P13637) |  |  |  |
|  |  | EHD3 (Q9NZN3) |  |  |  |
|  |  | ACTC1 (P68032) |  |  |  |
|  |  | TPRG1L (Q5T0D9) |  |  |  |
|  |  | LRP2 (P98164) |  |  |  |
|  |  | ANXA5 (P08758) |  |  |  |
|  |  | FLNB (O75369) |  |  |  |
|  |  | PDCD6IP (Q8WUM4) |  |  |  |
|  |  | APRT (P07741) |  |  |  |
|  |  | RAB8A (P61006) |  |  |  |
|  |  | CHP1 (Q99653) |  |  |  |
|  |  | GBE1 (Q04446) |  |  |  |
|  |  | ATP6V1A (P38606) |  |  |  |
|  |  | STXBP1 (P61764) |  |  |  |
|  |  | COX4I1 (P13073) |  |  |  |
|  |  | ATP6V1G1 (O75348) |  |  |  |
|  |  | ARF1 (P84077) |  |  |  |
|  |  | ACAT1 (P24752) |  |  |  |
|  |  | CAPZA1 (P52907) |  |  |  |
|  |  | QSOX1 (O00391) |  |  |  |
|  |  | TUBA1A (Q71U36) |  |  |  |
|  |  | NPEPPS (P55786) |  |  |  |
|  |  | PSMA2 (P25787) |  |  |  |
|  |  | CAPN5 (O15484) |  |  |  |
|  |  | NAP1L4 (Q99733) |  |  |  |
|  |  | ACO1 (P21399) |  |  |  |
|  |  | YES1 (P07947) |  |  |  |
|  |  | DYNC1H1 (Q14204) |  |  |  |
|  |  | SRI (P30626) |  |  |  |
